# Supplementary material for: Community-based rehabilitation training after stroke: results of a pilot randomised controlled trial (ReTrain) investigating acceptability and feasibility
Source: BMJ Open. 2018 Feb 15;8(2):e018409. doi: 10.1136/bmjopen-2017-018409 (PMC5829775; doi:10.1136/bmjopen-2017-018409)
Supplement: Author's manuscript [file bmjopen-2017-018409.draft_revisions.pdf]

BMJ Open

BMJ Open is committed to open peer review. As part of this commitment we make the peer review history of every article we publish publicly available.

When an article is published we post the peer reviewers' comments and the authors' responses online. We also post the versions of the paper that were used during peer review. These are the versions that the peer review comments apply to.

The versions of the paper that follow are the versions that were submitted during the peer review process. They are not the versions of record or the final published versions. They should not be cited or distributed as the published version of this manuscript.

BMJ Open is an open access journal and the full, final, typeset and author-corrected version of record of the manuscript is available on our site with no access controls, subscription charges or pay-per-view fees (http://bmjopen.bmj.com).

If you have any questions on BMJ Open's open peer review process please email editorial.bmjopen@bmj.com

BMJ Open

Community-based Rehabilitation Training after stroke: Results of a pilot randomised controlled trial (ReTrain)

Journal:	*BMJ Open*
Manuscript ID	bmjopen-2017-018409
Article Type:	Research
Date Submitted by the Author:	28-Jun-2017
Complete List of Authors:	Dean, Sarah; PenCLAHRC University of Exeter Medical School,<br>Poltawski, Leon; University of Exeter Medical School<br>Forster, Anne; University of Leeds, Academic Unit of Elderly Care and Rehabilitat<br>Taylor, Rod; University of Exeter, Peninsula Medical School<br>Spencer, Anne<br>James, Martin; South West NHS Trust<br>Allison, Rhoda; Torbay and Southern Devon Health and Care NHS Trust<br>Stevens, Shirley; University of Exeter Medical School<br>Norris, Meriel; Brunel University London<br>Shepherd, Anthony; University of Portsmouth, Sport and Exercise Science<br>Lando, Paola; University of Exeter Medical School<br>Pulsford, Richard; University of Exeter, Sports and Health Science<br>Hollands, Laura; University of Exeter Medical School<br>Calitri, Raff; University of Exeter Medical School
**Primary Subject Heading**:	Rehabilitation medicine
Secondary Subject Heading:	Cardiovascular medicine, Health services research
Keywords:	STROKE MEDICINE, REHABILITATION MEDICINE, Clinical trials < THERAPEUTICS

SCHOLARONE™
Manuscripts

Title Community-based Rehabilitation Training after stroke: Results of a pilot randomised controlled trial (ReTrain)

Sarah G Dean[1], Leon Poltawski[1], Anne Forster[2], Rod S Taylor[1], Anne Spencer[1], Martin James[1,3], Rhoda Allison[4], Shirley Stevens[1], Meriel Norris[5], Anthony I Shepherd[6], Paolo Landa[1], Richard M Pulsford[7], Laura Hollands[1], Raff Calitri[1].

[1]University of Exeter Medical School, Exeter, EX1 2LU, UK.

[2]Academic Unit of Elderly Care and Rehabilitation, University of Leeds, Bradford, BD9 6RJ, UK.

[3]Royal Devon & Exeter Hospital, Exeter, EX2 5DW, UK.

[4]Torbay and South Devon NHS Foundation Trust, Torquay, TQ2 7TD, UK.

[5]Department of Clinical Sciences, Brunel University London, Middlesex UB8 3PH, UK.

[6] Department of Sport and Exercise Science, University of Portsmouth, Portsmouth, PO1 2ER.

[7]Sports & Health Sciences, University of Exeter, Exeter, EX1 2LU, UK.

Corresponding author

Associate Professor Sarah Dean,

Psychology Applied to Rehabilitation and Health,

University of Exeter Medical School,

College House

St Luke's Campus,

Exeter,

EX1 2LU,

Email: s.dean@exeter.ac.uk

01392 722984

Word count 4267

For peer review only - http://bmjopen.bmj.com/site/about/guidelines.xhtml

Key words: Stroke, Rehabilitation Medicine, Clinical Trials.

Abstract

Objectives: To assess acceptability and feasibility of trial processes and the ReTrain intervention including an assessment of intervention fidelity. **Design:** A two-group, assessor-blinded, randomised controlled trial with parallel mixed methods process and economic evaluations**. Setting:** Community settings across two sites in Devon. **Participants**: Eligible participants were: 18 years old or over with self-reported mobility issues, no contraindications to physical activity, discharged from NHS or any other formal rehabilitation programme at least 1 month prior to entry into the trial, willing to be randomised to either control or ReTrain and to attend the training venue, possessing cognitive capacity and communication ability sufficient to participate in the study. Participants were individually randomised (1:1) via a computer generated randomisation sequence minimised for time since stroke and level of functional disability. Only outcome assessors independent of the research team were blinded to group allocation. **Interventions:** ReTrain comprised (1) an introductory one-to-one session; (2) ten, twice weekly group classes with up to two trainers and eight clients; (3) a closing one-to-one session, followed by three drop-in sessions over the subsequent three months. Participants received a bespoke home-based training programme. All participants received treatment as usual. The control group received an exercise after stroke advice booklet. **Outcome measures:** Candidate primary outcomes included functional mobility and physical activity. **Results:** Forty-five participants were randomised (ReTrain=23; Control=22); data were available from 40 participants at six months follow-up (ReTrain=21; Control=19) and 41 at nine months follow-up (ReTrain=21; Control=20). We demonstrated ability to recruit and retain participants. Participants were not burdened by the requirements of the study. We were able to calculate sample estimates for candidate primary outcomes and test procedures for process and health economic evaluations. **Conclusions:** All objectives were fulfilled and indicated that a definitive trial of ReTrain is feasible and acceptable.

Registration: ClinicalTrials.gov: trial number NCT02429180.

Funding: The Stroke Association TSA 2014-13

For peer review only - http://bmjopen.bmj.com/site/about/guidelines.xhtml

Strengths and limitations of this study

- A community-based exercise intervention after stroke developed with service users
- A pilot randomised trial developed following MRC guidelines on complex interventions
- Mixed-method approach for answering feasibility and acceptability objectives
- Objective physical activity capture via accelerometry but subjective self-report outcomes for functional mobility and psycho-social measurements

For peer review only

For peer review only - http://bmjopen.bmj.com/site/about/guidelines.xhtml

Introduction

Five years after initial stroke, one in three individuals have residual physical impairment[1]. This equates to over 300,000 individuals in the UK living with disability from stroke[2]. Provision of stroke rehabilitation is typically front loaded, with resources focussed on in-patient care and early supported discharge. Support tapers off after a few months[3] with many individuals reporting unmet long-term needs[4].

The National Clinical Guideline for Stroke advise for secondary prevention that stroke survivors engage in 150 minutes of physical activity a week, in bouts of 10 minutes or more, starting light and developing across time to moderate levels of intensity[5]. However, many stroke survivors do not meet these recommendations[6 7] due to combinations of personal (e.g., physical or psychological impairments) and environmental factors (e.g., lack of programmes and facilities). To address this problem, community-based programmes are promoted[8-10]. These tend to focus on cardiovascular fitness with less emphasis on functional improvements or on promoting on-going exercise self-management. National stroke guidelines[5] identify the importance of interventions for functional improvement[11] and self-management[12] but evidence is lacking regarding these types of intervention[13].

Action for Rehabilitation following Neurological Injury (ARNI) is an approach aimed at improving function and facilitating self-management[14] and has a detailed self-help book. The ARNI approach embodies a set of principles (e.g. instilling a commitment to regular exercise) and techniques tailored to individual need. The ARNI Institute trains registered exercise professionals to deliver key ARNI techniques. Clinical Commissioning Groups (CCGs), charitable, and local authorities have started to provide community-based ARNI training for stroke survivors, which has been positively received by participants, carers and practitioners[15], however there is currently no randomised controlled trial (RCT) evidence for evaluating its impact on stroke outcomes or its cost-effectiveness.

Background and objectives

Using the Medical Research Council's framework for the development and evaluation of complex interventions[16] and considerable Patient and Public Involvement we have designed a testable programme called Rehabilitation Training (ReTrain)[17-20]. ReTrain is a community-based, manualised group programme combining ARNI principles and key techniques with best practice guidelines for stroke[9 17]. The overall aim of our pilot RCT was to inform the design and delivery of a definitive RCT. Our objectives were to: 1) assess feasibility and acceptability of recruitment, randomisation, allocation concealment and outcome assessment blinding; 2) determine retention rates; 3) check ReTrain's acceptability and feasibility for participants, and refine the Trainer Manual; 4) test candidate outcome measures, assess their burden, levels of completion, and estimate outcome variance (to inform definitive trial sample size); 5) perform process evaluation including intervention

For peer review only - http://bmjopen.bmj.com/site/about/guidelines.xhtml

fidelity assessment; 6) calculate ReTrain costs and assess feasibility of collecting health and social service resource use.

Methods

A brief methods overview is provided in accordance with guidance for reporting pilot trials[21]; further details are available in the published protocol[22].

Trial design

ReTrain was a two-group, assessor-blinded, randomised controlled external pilot trial with parallel mixed methods process and economic evaluations. Eligible participants were individually randomised 1:1 to intervention (ReTrain) or control (exercise advice booklet[23]).

Participants

Inclusion criteria were: i) diagnosis of stroke; ii) at least 1 month since discharge from NHS physical rehabilitation services; iii) able to walk independently indoors with or without mobility aids, but with self-reported difficulty with stairs, slopes or uneven surfaces; iv) willingness to be randomised and to attend the training venue; v) cognitive capacity and communication ability sufficient to participate.

Exclusion criteria were: less than 18 years old, currently (or within one month of) receiving ARNI training or have contraindications to moderate to vigorous physical activity (adapted from American College of Sports Medicine guidelines[24]). Participants were recruited from two CCGs. Participants were identified by: (1) clinicians in NHS primary care, hospital and community stroke services; (2) contacts in the local Clinical Research Network and Clinical Research Facility; (3) promotion via local stroke support networks (e.g. Stroke Association); (4) word of mouth, study flyers and adverts.

Intervention

ReTrain aims to: (1) enhance function through task-related practice, teaching compensatory techniques, and providing targeted strength training (cardiovascular fitness gains also occur through these activities); (2) develop self-management skills for on-going rehabilitation; (3) deliver personalised training using negotiated goals and (4) instil a commitment to regular exercise for health improvement and longer-term maintenance. ReTrain facilitates safe and efficient practice of walking in varied terrains, kerbs, cambers and in crowds, turning and moving quickly, climbing steps and stairs without rails, getting to and from the floor without furniture or other aids, and moving without mobility aids or while carrying loads. Training is based on a manual and led by personal trainers on the UK Register of Exercise Professionals (level 3 or above) who are ARNI-trained and accredited and have had additional training in the delivery of ReTrain. There was a maximum ratio of one trainer to four stroke survivors. ReTrain was delivered in a community setting (one gym, two church halls, one community centre) with twice weekly two hour sessions over three months, comprising: an introductory

For peer review only - http://bmjopen.bmj.com/site/about/guidelines.xhtml

one-to-one session (home visit); ten, twice-weekly group classes with up to two trainers and eight clients (training venue); a closing one-to-one session (home visit); followed by three (one per month) drop-in sessions. Participants completed bespoke home-based training (homework) throughout.

Control

All participants received treatment as usual. The control group also received an advice booklet about exercise after stroke[23].

Outcomes

Feasibility, acceptability and process outcomes: numbers and details of those approached; recruitment and retention figures. ***Acceptability of randomisation, outcome measurement burden, and the intervention:*** completion of questionnaires and objective assessments; interviews with ten intervention and ten control group members, and the trainers. ***Safety****:* Adverse events[25] identified via trainer and ReTrain participants (during the programme) and participant reports (all participants during 6 and 9-month assessments). ***Intervention fidelity:*** attendance registers, accelerometry, exercise 'homework' diaries, trainer completed session checklists and video analysis of (early, middle and late programme) training sessions.

We tested a range of candidate primary and secondary outcome measures. ***Primary Outcomes***: Rivermead Mobility Index[26 27]; Timed Up and Go Test[28]; modified Patient-Specific Functional Scale[29]; 7-day objective physical activity levels using wrist-worn accelerometry (GENEActiv, Activinsights, Kimbolton, Cambridge UK) and a physical activity diary. ***Secondary Outcomes***: Stroke Self-efficacy Questionnaire[30]; Fatigue Assessment Scale[31 32], exercise beliefs and exercise self-efficacy questionnaires[33], SF12[34], EQ-5D-5L[35], Stroke Quality of Life (QoL) questionnaires[36], Carer Burden Index[37] and Health and Social Service use through a Service Receipt Inventory[38].

Physical outcome baseline assessments (completed by research team) and follow-up assessments (at 6 and 9-months, completed by blinded assessor) were conducted in the participant's home. Researchers visited participants to fit the accelerometer, drop off questionnaires and diary one week prior to blind assessor visits. Assessors administered primary outcome physical measures and collected accelerometers, questionnaires and diaries.

Sample size

We required 48 participants (24 per group) as (a) 30 complete data sets are recommended for pilot studies to estimate outcome variance[39] and (b) we wanted to investigate variations in context by running the intervention three times (*i.e.* 3 x 8 patients). This number also

For peer review only - http://bmjopen.bmj.com/site/about/guidelines.xhtml

allowed estimation of a predicted attrition rate of 20% with a precision of ± 5% with 95% certainty.

Randomisation and blinding

The random sequence was computer generated with minimisation for time since stroke (≤ 3 months versus > 3 months) and level of functional disability (modified Rankin Scale (mRS)[40] score ≤ 2 versus > 2). Allocation concealment was ensured by using a password protected validated web-based remote randomisation service. The Trial Manager requested randomisation only after a cohort of participants had been consented.

Participants, trainers providing the intervention, and researchers conducting the process and economic evaluations could not be blinded to allocation. However, outcomes were assessed by independent researchers (not based at research centre) who were blinded to group allocation. Participants were reminded not to reveal their allocation to assessors but any un-blinding was recorded; after assessments assessors were asked to guess participant allocation.

Data Analysis

Analysis was primarily descriptive with participant flow summarised and estimates of screening, recruitment and attrition reported. Means and standard deviations for all outcomes are reported at baseline, 6 and 9-months follow-up for each group.

Intervention fidelity was assessed using mixed methods: qualitative video analysis comparing the Trainer Manual standard versus observed technique (two researchers independently assessed videos) combined with interview data and summary scores from trainer completed session checklists. Qualitative data were analysed descriptively and with content analysis; additional thematic analysis was used for interview data.

We used a micro-costing approach to calculate costs associated with ReTrain: staff time (trainers, administrator, facilitators), venue hire, training equipment (annualised over time), course materials, consumables, travel costs (participants, trainers and facilitators). We analysed the relative benefits of calculating health related QoL using SF-6D (developed from the SF-12) over the QALY calculated (using EQ-5D 5L) from the baseline measures.

Sample size estimates for a definitive trial were calculated for candidate primary outcomes using the standard deviation observed in this pilot population and published minimal clinical important difference (MCID) at 90% power and 5% alpha, and assuming 20% attrition. Where no published MCID could be sourced, we assumed a small to moderate effect size of 0.4 of a standard deviation[41]. The trial statistician undertook calculations using the 'samspi' command in STATA v14.2

Results

For peer review only - http://bmjopen.bmj.com/site/about/guidelines.xhtml

Recruitment took place from June 2015 to January 2016. The intervention ran in four cohorts, participant flows are shown for each (Figure 1a) and for the trial overall (Figure 1b). Initial recruitment was slow so to prevent late running of the trial we split the first cohort. Six-month follow-up outcome assessments took place January to July 2016 and 9-month follow-up April to October 2016.

Objective 1: *Assess the feasibility and acceptability of recruitment, randomisation, allocation concealment and processes for outcome assessment and blinding*

We screened 115 individuals to recruit 50 participants (Figure 1a) in 8 months (2 months ahead of schedule). Of these, 45 (90%) were randomised (Figure 1a and 1b). Five individuals withdrew prior to randomisation due to ill health or the time lag between agreeing to take part and a cohort being ready to randomise. Table 1 shows baseline characteristics of those randomised, indicating a balance of characteristics across trial arms.

Blinding of outcome assessors was considered successful as only 2/41 (5%) participants revealed their allocations after completion of outcome measures, both were intervention participants. Different assessors were used for subsequent assessments therefore risk of bias was minimised.

Table 1 Baseline participant demographics

	ReTrain (N= 23)	Control (N=22)
**Gender, n**		
Male (%)	16 (70%)	14 (67)
**Age (years): mean**	70	71
**Age Category (N=45): n (%)**		
<45	1 (4%)	0 (%)
46-50	0 (0%)	1 (5%)
51-60	3 (13%)	2 (9%)
61-70	10 (43%)	6 (27%)
71-80	5 (22 %)	8 (36%)
81-90	2 (9%)	5 (23%)
90+	2 (9%)	0 (0%)
**Time Since Stroke (no. months):**		
< 12	3 (13%)	3 (14%)
12-24	4 (17%)	4 (18%)
25-48	5 (22%)	5 (23%)
49-72	2 (9%)	5 (23%)
73-96	4 (17%)	2 (9%)
97+	5 (22%)	3 (14%)
**Time Since Stroke Minimisation Categories (months):n, (%)**		
<=3 months	1 (4%)	0 (0%)
>3 months	22 (96%)	22 (100%)

For peer review only - http://bmjopen.bmj.com/site/about/guidelines.xhtml

**Type of Stroke, n (%)**		
Haemorrhagic	3 (13%)	1 (5%)
Ischaemic	15 (65%)	15 (68%)
Both	0 (0%)	1 (5%)
Missing	5 (22%)	5 (23%)
**Stroke Rehabilitation (weeks):**		
n,<br>Average no. weeks	21,<br>7	21,<br>14
Median no. weeks	6	12
Range	0-32	0-88
Unknown length rehab: n	2	1
**Functional Disability (Simplified Modified Rankin Scale score- sMRS): n (%)**		
0	1 (4%)	0 (0%)
1	2 (9%)	1 (5%)
2	4 (17%)	9 (41%)
3	16 (70%)	12(55%)
**sMRS minimisation categories: n(%)**		
<=2	7 (30%)	10 (45%)
>2	16 (70%)	12 (55%)
**Co-morbidities^, n (%)**		
Hypertension	18 (78%)	18 (82%)
Type 2 Diabetes Mellitus	4 (17%)	4 (18%)
Depression	8 (35%)	5 (23%)
Chronic Kidney Disease	2 (9%)	1 (4%)
Asthma / COPD	4 (17%)	3 (14%)
Other	5 (22%)	3 (14%)
**Medications^, n (%)**		
Diuretics	3 (13%)	1 (5%)
Anticoagulants	8 (35%)	10 (45%)
Antiplatelet	15 (65%)	12 (55%)
Antihypertensives<br>Calcium Channel Blockers<br>ACE inhibitors<br>Other	 6 (26%)<br>13 (57%)<br>9 (39%)	 14 (64%)<br>8 (36%)<br>7 (32%)
Statins	18 (78%)	19 (86%)
Anti-depressants	8 (35%)	5 (23%)
Diabetes medication	4 (17%)	4 (18%)
Chronic pain medication	12 (52%)	8 (36%)
Other	5 (22%)	3 (14%)
**Employment Status, n (%)**		
Employed (and working)	2 (9%)	1 (5%)
Retired	18 (78%)	15 (68%)
Semi-retired	1 (4%)	0 (0%)
Unemployed	2 (9%)	5 (27%)
**Pre-stroke Exercise History, n**		
Exerciser (%)	10 (43%)	8 (36%)
**Mini Mental State Exam:**		

For peer review only - http://bmjopen.bmj.com/site/about/guidelines.xhtml

n,<br>Mean (SD)	22*,<br>27.5 (2.54)	22,<br>27.9 (3.01)
Median	28	29
Range	19– 30**	19-30**

^ Participants may have more than one co-morbidity / medication.
*1 participant with severe aphasia had difficulties completing the MMSE. The participant could understand and follow instructions and was considered cognitively able to participate in the trial.
**Higher scores indicate better cognitive function. Participants range from no to moderate degree of cognitive impairment.

Objective 2: *Acquire retention rates and outcome variance*

Forty out of 45 (88%, 95% CI: 76% to 96%) completed 6-month and 9-month follow-ups. Despite fewer people being randomised than expected, high retention preserved the number of datasets needed to perform our sample size estimates (Table 2).

Table 2 Sample estimates for potential candidate primary outcomes from ReTrain pilot RCT

Primary Outcome Measure	Sample Size Required
Rivermead Mobility Index	36 – 44*
Timed Up and Go	108 – 1962*
Modified Patient Specific Functional Scale	16 – 200*
Physical Activity (Accelerometer)	430*

*Sample size for 1:1 allocation at 90% power and 5% alpha and assuming 20% attrition given range of observed SDs observed in this trial and MCIDs

Objective 3: *Acceptability and feasibility for participants and complete the Trainer manual*

Eleven themes from 20 qualitative interviews summarise participants' views, Table 3 provides illustrative quotes.

a) Study Information

Participants considered information received as adequate. Five noted that information was limited, but most were unconcerned. Two added that too much information may have been detrimental to recruitment. Four others were satisfied with the information they received.

b) Outcome Measure Burden

Participants found the assessment process acceptable. Fifteen indicated no burden. Three participants indicated that the questionnaires placed burden on their carer.

For peer review only - http://bmjopen.bmj.com/site/about/guidelines.xhtml

For peer review only

Table 3 Participant quotes from qualitative interviews

Acceptability

"It is ten weeks, you do it twice a week. Personally for the first say three or four weeks, I'd think well this is getting me nowhere, but then you think that you notice things, things are improving and at the end of ten weeks you want to go for twenty weeks (4:119-125)

"I'd tell them [another stroke survivor] to go ahead and do it and to take it step by step and not to worry about it. Because you are treated with great respect, it was wonderful and they were. I'll never be able to speak highly enough of them." (25:388-390)

Intervention approach

"It opened my eyes to what can be done you know. How can I put it? It wasn't as if I believed that I couldn't do something it was being pointed in the right direction…heh I can do it…Great you've done it, you did it and you do it again. Yeah it was great" (4:358-361). "It wasn't easy at first, but I used to manage it" (5:246)

"It was the way they addressed how you do your exercises. What it is doing to you and all the rest of it. Now to me that was absolutely important, because it made sense of why you are doing all this pumping up and down, and if you can't do that, do this." (22:252-255)

"It was you felt as if you were a human being with them. You know and you were treated with respect…and although you couldn't do things and you felt a bit of an idiot, they never let you feel like that" (25:567-572).

"It's a bit like playing scales…it's not creative but as I gradually realise it, it could potentially be creative…doing something that I had been doing without thinking before and now couldn't. …Now and again I walk without my stick without realising it, that's creative I think." (6:354-392)

Impact of programme - psychologically

"I suppose it is attitude of mind as much as anything. I mean I felt I'd gone through that stage of training and that I was going to get better. It built my spirit up…I felt as if it, well it was worth the three months you know and at the end of the day I hope I'm going to get back to something like normal" (16:358-365)

"It really helped me mentally, you know I thought right I can do this because before I was going into my shell, thinking I can't do this and I can't do that. Oh I am not going out. Then I went on that [ReTrain] and it gave me an element of confidence." (43:562-565).

Impact of programme – physically

"you started to notice they are actually starting to fall into place. I don't remember doing that last time. But I am doing it now great get on with it I am doing it faster now" (4:189-190)

"I know if I went down which I did one day in the hall in the early stages of coming back home and I did manage to get up and walk upstairs…but I wouldn't have been able to do that had I not had that [training]" (16:475-477)

Homework adherence

"trainers were always on about doings exercises at home…I could never pin him down to how long that should be for though" (6; line 624).

Programme technique adherence

"I think that was the big thing you saw the benefits after the second, well the first or second session we had. 'Oh we can do that!'…even if you were chastised, 'come on get down, you have got to get up; you can do it!' you know 'try this'. There was a little trick which was another little thing of getting up from the ground which was, didn't need any strength whatsoever and that was one of the big major things, especially for this other guy…doing it in a simple way of you know just sliding around and getting up using your own motion of getting up…that momentum is fantastic, lots of little things like that and you could see it worked." (22:479-499).

For peer review only - http://bmjopen.bmj.com/site/about/guidelines.xhtml

c) Venue

Half of the ReTrain participants were very positive about the training venues. Important features were: space, provision of fluids (water, tea), easy availability of parking. For some the travelling distance was a concern; two noted their venue (a gym) was very noisy, insufficiently heated and the session time was too early. Some noted the small amount of equipment as an advantage (it aided transfer of exercises to their home) whereas others felt the equipment was not sufficiently specialist.

d) Adherence to ReTrain (see also Objective 5)

All ten ReTrain interviewees reported training in the five core (ARNI) techniques. Homework was discussed by all but lacked specificity, only two had clear homework examples that were effectively incorporated into their training. Although goal setting was a core element, only four specifically identified how their goals were linked into their overall programme. Three participants reported not attending drop-in sessions due to lack of information. Of three who attended two suggested the drop-ins repeated previous sessions.

e) Group dynamics

Group working was positively regarded and seen as integral to programme effectiveness. There were exceptions, one participant did not find 'performing' in public a positive experience. Likewise some suggested that groups reduced training intensity relative to one-to-one training.

f) Co-morbidities

Participants identified several co-morbidities e.g.: knee replacements, cancer, angina, diabetes, amputation and depression. These had potential to impact on both the training and research participation but for most any concerns were accommodated by trainers. However, in one case some uncomfortable discussions occurred before an appropriate balance of perceived capability and training challenges was reached. Three participants with visual deficits, dyslexia and dysgraphia mentioned difficulties completing the research documents.

g) Carer Health

Two ReTrain participants commented on how commitment to the programme impacted on their partner's health: one stopped attending sessions because the time away resulted in excessive strain on his wife; another expressed similar concern but did not stop attending.

h) Trainer Manual

We refined the Trainer Manual throughout the study. Issues raised during interviews guided revisions including greater emphasis and clarification about use of goal setting, drop-ins, homework diaries, and managing participants with co-morbidities.

Objective 4: *Assess outcome completion and burden*

For peer review only - http://bmjopen.bmj.com/site/about/guidelines.xhtml

For peer review only

We collected baseline (n=41), 6-month (n=40) and 9-month (n=41) follow-up data on the majority of participants (Figure 1b). Accelerometry wear time (24 hours for 7 days) was high, most having 6 or more valid days (≥ 16hrs per day, including ≥ 1 weekend day). Only two participants at baseline, one at 6-months and three at 9-months did not achieve 4 valid days of wear time. There was very little missing data. For three primary outcome measures there was only one participant with missing data at any given assessment time-point. For secondary outcomes there was either no missing data or only one to two participants with missing data at each time-point, apart from the exercise diary (between two and four participants with missing data at each time-point) and the Service Receipt Inventory (between three and seven participants with missing data). There were eight participants without accelerometer data at 9-month assessment owing to hardware (device) and software (data extraction method) malfunctions.

Objective 5: *Perform process evaluation with an assessment of intervention fidelity*

We implemented a comprehensive video recording schedule (over 200 recordings) to capture participant and trainer adherence to key ARNI techniques. Both trainers and participants demonstrated high adherence. Modifications to techniques (to accommodate participant co-morbidities) were captured and informed Trainer Manual development.

We combined metrics from attendance registers and homework records to generate a 'dose'/adherence score, categorising individuals into low (< 50%), medium (50% - 75%) and high (>75%) adherence categories. Of 23 ReTrain participants two did not receive the intervention (one returned to work; one withdrew from study), five had low adherence, five medium adherence, and eleven high adherence. These latter 16 (70%) were considered to have received sufficient 'dose' of ReTrain.

Trainers varied in their completion of session checklists: pre-exercise and end-of-session components were less consistently reported compared to ARNI techniques but overall there was good adherence to programme delivery.

Objective 6: *Calculate the cost of intervention delivery and feasibility of collecting health and social service resource use.*

ReTrain costs were generated for each cohort, accounting for different programme sizes (four or eight participants) and venues. Costs per participant ranged from £615 to £972. The total per participant cost for ReTrain (assuming 24 participants) was £777. We conducted medical notes review on 35/41 participants and compared this 'gold standard' with self-reported health resource use. Participants reported using fewer resources compared to case notes review. Data from medical notes informed the cost-utility and effectiveness frameworks for use in a definitive trial.

Descriptive analysis of participant outcomes

Primary and secondary outcome data are summarised in Table 4a and 4b respectively.

For peer review only - http://bmjopen.bmj.com/site/about/guidelines.xhtml

Table 4a: Means and standard deviations as a function of trial arm and measurement time point for candidate primary outcome measures in the ReTrain pilot trial

	Data collection time point					
	Baseline		6 month^		9 month^	
**Measures:** n, Mean (SD)	**ReTrain (N=23)**	**Control (N=22)**	**ReTrain (N=21)**	**Control (N=20)**	**ReTrain (N=21)**	**Control (N=20)**
Rivermead Mobility Index	23, 11.41 (3.05)	22, 11.68 (2.23)	21, 12.14 (2.73)	19, 12.47 (1.87)	21, 12.24 (3.27)	20, 12.65 (1.81)
Modified Patient Specific Functional Scale	22, 2.95 (1.85)	22, 2.55 (1.23)	21, 3.47 (2.12)	19, 3.56 (1.69)	21, 3.25 (2.03)	20, 3.74 (1.86)
Timed Up and Go (secs)	23, 27.57 (27.57)	21, 21.24 (11.18)	21, 20.76 (19.64)	19, 16.37 (9.69)	21, 20.76 (19.25)	20, 15.95 (12.00)
Physical Activity: **Diary***	21, 6.67 (19.20)	20, 10.69 (17.39)	21, 17.39 (24.28)	19, 25.60 (34.98)	19, 13.92 (22.25)	20, 35.11 (49.70)
Physical Activity **(Accelerometer): Total PA minutes***	21, 145.10 (118.27)	20, 165.56 (139.09)	19, 134.88 (129.77)	18, 178.15 (155.07)	16, 152.08 (118.52)	17, 197.42 (144.31)
Physical Activity **(Accelerometer): Light PA minutes***	21, 92.78 (93.15)	20, 110.18 (115.65)	19, 95.67 (105.50)	18, 121.80 (122.46)	16, 99.33 (99.54)	17, 134.54 (126.36)
Physical Activity **(Accelerometer): MVPA PA minutes***	21, 52.32 (68.94)	20, 55.38 (39.66)	19, 39.21 (39.33)	18, 56.35 (51.26)	16, 52.75 (60.03)	17, 62.88 (41.73)
Physical Activity **(Accelerometer): Moderate PA minutes***	21, 50.53 (66.77)	20, 53.38 (37.04)	19 37.73 (37.40)	18, 53.07 (43.99)	16, 51.11 (58.54)	17, 60.93 (40.84)
Physical Activity **(Accelerometer): Vigorous PA minutes***	21, 1.79 (3.85)	20, 2.00 (3.96)	19, 1.48 (2.39)	18, 3.28 (8.14)	16, 1.64 (2.38)	17, 1.94 (2.33)

Note: ^ post randomisation; *Average minutes of physical activity per day

For peer review only

For peer review only - http://bmjopen.bmj.com/site/about/guidelines.xhtml

Table 4b: Means and standard deviations as a function of trial arm and measurement time point for candidate secondary outcome measures in the ReTrain pilot trial

	Data collection time point					
	Baseline		6 month^		9 month^	
**Measures:** n, Mean (SD)	**ReTrain (N=23)**	**Control (N=22)**	**ReTrain (N=21)**	**Control (N=20)**	**ReTrain (N=21)**	**Control (N=20)**
Fatigue Assessment Scale	23, 27.17 (7.14)	22, 25.49 (7.44)	21, 24.05 (6.26)	19, 24.05 (8.86)	20, 27.45 (5.95)	20, 25.83 (9.14)
Stroke Self-efficacy	22, 72.41 (22.00)	22, 73.46 (17.87)			20, 73.73 (19.63)	20, 74.40 (16.94)
Exercise Beliefs	23, 3.66 (0.70)	22, 3.78 (0.52)			19, 4.03 (0.59)	19, 3.73 (0.52)
Exercise Self-efficacy	23, 3.26 (0.92)	22, 3.32 (0.89)			19, 3.32 (0.89)	18, 3.22 (1.06)
Stroke Quality of Life (Total)	22, 3.31 (0.68)	22, 3.45 (0.69)			20, 3.38 (0.70)	20, 3.63 (0.82)
EQ-5D-5L	22, 0.51 (0.25)	20, 0.55 (0.24)			19, 0.52 (0.24)	20, 0.62 (0.25)
SF-12: Physical Component	21, 33.12 (7.22)	20, 31.83 (6.69)			19, 33.74 (6.44)	19, 33.25 (6.91)
SF-12: Mental Component	21, 50.10 (7.11)	20, 50.68 (7.98)			19, 50.47 (6.51)	19, 48.05 (8.45)
Carer Burden Index	8, 11.39 (8.03)	10, 7.40 (7.83)			9, 9.89 (7.22)	6, 9.50 (8.92)

Note: ^ post randomisation; grey cells indicate measurement not taken at this time point

For peer review only - http://bmjopen.bmj.com/site/about/guidelines.xhtml

Safety

During assessment periods there was one serious but unrelated event in the intervention group (none in the control group) and slightly less overall adverse events in the intervention group (Table 5a). For ReTrain only there were three adverse events at the training venue and a further three during the overall intervention period: four were unrelated, one possibly related, and one probably related; none were definitely related to the intervention (Table 5b).

Table 5a: Adverse Events (AE) and Serious Adverse Events (SAE) reported during 6 and 9-month outcome assessment periods for both ReTrain and Control group

	Event Type	Total Events	Attribution				N People Reporting Event
ReTrain (N=21)			Related	Probably Related	Possible Related	Unrelated	
	**AE**	125	6	5	73	41	19
	**SAE**	1	0	0	0	1	1
Control (N=20)	Event Type	Total Events	Related	Probably Related	Possible Related	Unrelated	N People Reporting Event
	**AE**	150	0	0	0	150	19
	**SAE**	0	0	0	0	0	0

Table 5b: Adverse Events (AE) and Serious Adverse Events (SAE) reported during ReTrain programme

	Event Type	Total Events	Attribution				N People Reporting Event
ReTrain (N=21)			Related	Probably Related	Possible Related	Unrelated	
	**AE**	22	7	0	12	3	11
	**SAE**	6	0	1	1	4	5

For peer review only - http://bmjopen.bmj.com/site/about/guidelines.xhtml

Discussion

The ReTrain pilot trial met all its pre-stated feasibility objectives: the intervention, trial design and research processes were acceptable to participants as well as feasible and safe to deliver; we demonstrated feasibility of recruitment and retention; participants were not unduly burdened by study requirements and there were high completion rates for most outcome measures. We also successfully rehearsed procedures for process and health economic evaluations as well as trial governance processes (trial management and independent trial steering meetings) and maintained our strong Patient and Public Involvement. Participant interviews, outcome measurement results and fidelity assessments highlighted refinements that we have already, or can, put in place for a future definitive RCT of ReTrain. Our trial compares favourably with another feasibility RCT assessing the delivery of the Bridges stroke self-management programme[42] which had relatively low recruitment, questions regarding programme delivery in addition to usual rehabilitation, and recommendations for further assessment of intervention fidelity. Some of their findings were similar to ReTrain: participants were broadly positive about their programme; health professionals found it acceptable to use and researchers noted the lack of outcome measure sensitivity for detecting change[42].

Limitations and lessons for planning design of a future trial

We have not identified a clear candidate primary outcome for a definitive RCT from this pilot work. Identifying robust outcome measures in rehabilitation trials is a common problem[43] compounded by variability in stroke related disability and participants' comorbidities. This pilot trial was not designed (statistically powered) to test for differences between treatment arms, so no inferential analyses were performed. Any perceived trend (or absence of a trend) should not be interpreted as an indication of an effect (or its absence) and outcomes should not be selected based upon any assumed trend. Acceptability outcomes coupled with a pragmatic and efficient (cost-effective) trial design better inform choice of outcome. From our sample the Timed Up and Go task would be unsuitable due to potentially large sample size requirements (~2000 participants) and the baseline high levels of mobility meant the Rivermead Mobility Index demonstrated a ceiling effect, so could only be used if we altered inclusion criteria. Physical activity was measured robustly via accelerometry and may be the best candidate. We had some software and hardware malfunctions but important lessons have been learned to mitigate these problems in future. Capture of frequency and intensity of activity would allow comparison with stroke guidelines. Although there is a cost implication, accelerometry provides a more objective measurement of daily activity and may also be an adequate proxy of functional mobility.

Further limitations relate to the lack of validation of our adherence measure and the local demographics: our sample did not have a wide age range or ethnic diversity. Whilst we did demonstrate delivery in different locations in the South West our plans for a larger definitive trial would include a wider demographic from more centres across the UK.

For peer review only - http://bmjopen.bmj.com/site/about/guidelines.xhtml

For a future trial we plan to implement more readable, higher quality written (and pictorial) information and questionnaires although the amount of information provided was appropriate. We will mitigate recruitment loss prior to randomisation by establishing expression of interest and eligibility to take part but delaying taking consent until we are confident of sufficient numbers to create a cohort for randomisation; this has resource implications that will need to be built into future funding. We will run ReTrain in community centres or halls as these were more acceptable and much cheaper than gyms; we will provide a more detailed ReTrain induction to ensure trainers understand and communicate all components of the programme. Revisions to the Trainer Manual have already been made. For the QALY comparisons recent policy changes mean the conversion from SF-12 to SF-6D has been phased out, and so less justification for using the SF-12 in a future study. Instead we will consider using the Stroke Impact Scale (SIS) as this is a valid health-related QoL measure. This may also be a better candidate self-report primary outcome measure for a definitive trial as it has shown sensitivity in long-term stroke survivors who have mild to moderate stroke[44]. The SIS assesses multiple facets of physical and emotional issues and so would align with perceived physical and psychological benefits participants attribute to ReTrain. Our sample size estimates for candidate primary outcomes (Table 2) indicate we will need a moderately sized trial (n=430) for physical activity assessed by accelerometry or a smaller trial (n=96) if we use the physical component domain of the SIS (based on 80% power, 5% alpha and assuming 20% attrition[45]). We have established appropriate process evaluation methods to capture multiple facets of intervention fidelity. Moreover we have some new insights into how to enhance delivery by trainers and engagement by participants (e.g. more focus on individually tailored goal setting; goal and homework reviews; better explanation and promotion of the drop-in sessions).

Generalisability

This pilot study was not designed to demonstrate generalisability however our participant population represent the sub-set of community-dwelling stroke survivors who have some independent mobility but remain with stroke-related disability that affects their QoL. Our participants also represent the growing proportion of people who have more than one long-term condition. ReTrain techniques target the effects of stroke but can accommodate other conditions which trainers take into account when preparing the participant's individually tailored programme. Some of the key ReTrain (ARNI based) techniques are designed for people with unilateral impairment, such as hemiparesis; however one of our participant's main unilateral impairment was due to diabetes related lower limb amputation, illustrating how ReTrain can accommodate people with multiple co-morbidities.

Conclusion

Our pilot trial has demonstrated that ReTrain is feasible, acceptable, and safe. We met our recruitment and retention targets and demonstrated our ability to run our intervention in different locations. Participants were not unduly burdened by study requirements and most

For peer review only - http://bmjopen.bmj.com/site/about/guidelines.xhtml

outcome measures had high levels of completion. We successfully tested procedures for process and health economic evaluations. Participant interviews, outcome measurement results and fidelity assessments highlighted some issues needing refinement prior to a future definitive RCT of ReTrain. Many of these have already been addressed and we intend to seek funding for a definitive trial.

Other information

Protocol Version: 5 Date: 20/04/2016. Published version available here: http://bmjopen.bmj.com/content/6/10/e012375.full

Acknowledgements

We thank our funders, the Stroke Association and the Peninsula Patient Involvement Group with the ReTrain Stroke Service User Group for their help. The National Institute for Health Research (NIHR) Collaboration for Leadership in Applied Health Research and Care South West Peninsula at the Royal Devon and Exeter NHS Foundation Trust also supported this work but views expressed are those of the author(s) and not necessarily those of the NHS, the NIHR or the Department of Health. We also thank our Trial Steering Committee: Ailie Turton (University of the West of England) Siobhan Creanor (Plymouth University), Debbie Neal (Bournemouth University), Justin Smallwood (Patient and Public representative), and Gail Seymour (University of Exeter - Sponsor).

Ethical review by NRES Committee South West – Cornwall & Plymouth (REC ref: 15/SW/04).

Author's Contributions

SGD led the team and drafted this article, RC prepared protocol, ethical submission and amendments, managed the project, contributed to analysis; LP drafted protocol prior to funding application, conducted interviews, contributed to analysis, AF, MJ, RA, MN, SGD & LP provided stroke rehabilitation expertise; RST provided statistical and trial methodological expertise, led analysis; MN provided qualitative expertise and analysed qualitative data; AIS led accelerometry work, supported by RP who provided accelerometry analysis; SGD & LP provided process evaluation expertise, SGD led the process evaluation and supervised LH; LH led video analysis work; AS provided health economic expertise and led economic work supported by PL; SS provided patient and public involvement expertise. All authors commented on the manuscript.

Data sharing statement: Participants did not consent for datasets to be stored or accessed outside of the research team. Therefore no datasets have been made publicly available.

Competing Interests: We declare funding from the Stroke Association.

For peer review only - http://bmjopen.bmj.com/site/about/guidelines.xhtml

References

1. Feigin VL, Barker-Collo S, McNaughton H, et al. Long-term neuropsychological and functional outcomes in stroke survivors: current evidence and perspectives for new research. *Int J Stroke* 2008;3(1):33-40.
2. Townsend N, Wickramasinghe K, Bhatnagar P, et al. Coronary heart disease statistics: 2012 edition. London: British Heart Foundation, 2012.
3. Teasell R, Mehta S, Pereira S, et al. Time to rethink long-term rehabilitation management of stroke patients. *Top Stroke Rehabil* 2012;19(6):457-62.
4. McKevitt C, Fudge N, Redfern J, et al. Self-reported long-term needs after stroke. *Stroke* 2011;42(5):1398-403.
5. Intercollegiate Stroke Working Party. National Clincial Guideline for Stroke, Fifth Edition. 5 ed. London: *Royal College of Physicians* 2016.
6. Rand D, Eng JJ, Tang PF, et al. How active are people with stroke?: use of accelerometers to assess physical activity. *Stroke* 2009;40(1):163-8.
7. Stroke Association. Stroke Statistics. London: Stroke Association, 2013.
8. Best C, van Wijck F, Dennis J, et al. A survey of community exercise programmes for stroke survivors in Scotland. *Health Soc Care Community* 2012;20(4):400-11.
9. NHS Stroke Improvement Programme. Life after stroke: Participating in community exercise and fitness 2012 [Available from: http://www.improvement.nhs.uk/stroke/CommunityStrokeResource/CSRLifeafterstroke/CSRLifeafterstrokeexercisepoststroke/tabid/226/Default.aspx accessed July 2012.
10. Harrington R, Taylor G, Hollinghurst S, et al. A community-based exercise and education scheme for stroke survivors: a randomized controlled trial and economic evaluation. *Clin Rehabil* 2010;24(1):3-15.
11. French B, Thomas LH, Leathley MJ, et al. Repetitive task training for improving functional ability after stroke. *Cochrane Database Syst Rev* 2007(4):Cd006073.
12. Lennon S, McKenna S, Jones F. Self-management programmes for people post stroke: a systematic review. *Clin Rehabil* 2013;27(10):867-78.
13. Brazzelli M, Saunders David H, Greig Carolyn A, et al. Physical fitness training for stroke patients. *Cochrane Database Syst Rev* 2011; (11). http://www.mrw.interscience.wiley.com/cochrane/clsysrev/articles/CD003316/frame.html.
14. Balchin T. The Successful Stroke Survivor: A new guide to functional recovery from stroke. Lingfield, UK: ARNI Trust 2011.
15. Poltawski L. Survey of Group ARNI classes in England, 2011, unpublished eport.
16. Craig P, Dieppe P, Macintyre S, et al. Developing and evaluating complex interventions: the new Medical Research Council guidance. *BMJ* 2008;337:a1655.
17. Poltawski L, Abraham C, Forster A, et al. Synthesising practice guidelines for the development of community-based exercise programmes after stroke. *Implement Sci* 2013;8:115.
18. Kilbride C, Norris M, Theis N, et al. Action for Rehabilitation from Neurological Injury (ARNI): A pragmatic study of functional training for stroke survivors. *Open J Ther Rehabil* 2013;1:40-51.
19. Poltawski L, Briggs J, Forster A, et al. Informing the design of a randomised controlled trial of an exercise-based programme for long term stroke survivors: lessons from a before-and-after case series study. *BMC Res Notes* 2013;6:324.

For peer review only - http://bmjopen.bmj.com/site/about/guidelines.xhtml

20. Norris M, Kilbride C, Mohagheghi A, et al. A Qualitative Exploration of Participation in an Exercise Instructor Led Functional Training Programme for Community Dwelling Stroke Survivors. *Int J Ther Rehabil* 2013;20(12):597-605.
21. Eldridge SM, Chan CL, Campbell MJ, et al. CONSORT 2010 statement: extension to randomised pilot and feasibility trials. *BMJ* 2016;355.
22. Dean SG, Poltawski L, Forster A, et al. Community-based Rehabilitation Training after stroke: protocol of a pilot randomised controlled trial (ReTrain). *BMJ Open* 2016;6(10).
23. Stroke Association. Exercise and stroke London2013 [Available from: https://www.stroke.org.uk/sites/default/files/exercise_and_stroke.pdf accessed 24 05 2017 2017.
24. ACSM. American College of Sports Medicine Guidelines for Exercise Testing and Prescription. Philadelphia: American College of Sports Medicine, 2005.
25. Carnes D, Mullinger B, Underwood M. Defining adverse events in manual therapies: A modified Delphi consensus study. *Man Ther* 2010;15(1);2-6.
26. Green J, Forster A, Young J. A test-retest reliability study of the Barthel Index, the Rivermead Mobility Index, the Nottingham Extended Activities of Daily Living Scale and the Frenchay Activities Index in stroke patients. *Disabil Rehabil* 2001;23(15):670-6.
27. Hsieh CL, Hsueh IP, Mao HF. Validity and responsiveness of the rivermead mobility index in stroke patients. *Scand J Rehabil Med* 2000;32(3):140-2.
28. Ng SS, Hui-Chan CW. The timed up & go test: its reliability and association with lower-limb impairments and locomotor capacities in people with chronic stroke. *Arch Phys Med Rehabil* 2005;86(8):1641-7.
29. Stratford P, Gill C, Westaway M, et al. Assessing Disability and Change on Individual Patients: A Report of a Patient Specific Measure. *Physiother Can* 1995;47(4):258-63.
30. Jones F, Partridge C, Reid F. The Stroke Self-Efficacy Questionnaire: measuring individual confidence in functional performance after stroke. *J Clin Nurs* 2008;17(7b):244-52.
31. Michielsen HJ, De Vries J, Van Heck GL. Psychometric qualities of a brief self-rated fatigue measure: The Fatigue Assessment Scale. *J Psychosom Res* 2003;54(4):345-52.
32. Mead G, Lynch J, Greig C, et al. Evaluation of fatigue scales in stroke patients. *Stroke* 2007;38(7):2090-5.
33. Shaughnessy M, Resnick BM, Macko RF. Reliability and validity testing of the short self-efficacy and outcome expectation for exercise scales in stroke survivors. *J Stroke Cerebrovasc Dis* 2004;13(5):214-9.
34. Bohannon RW, Maljanian R, Lee N, et al. Measurement properties of the short form (SF)-12 applied to patients with stroke. *Int J Rehabil Res* 2004;27(2):151-4.
35. Herdman M, Gudex C, Lloyd A, et al. Development and preliminary testing of the new five-level version of EQ-5D (EQ-5D-5L*). Qual Life Res* 2011;20(10):1727-36.
36. Williams LS, Weinberger M, Harris LE, et al. Development of a stroke-specific quality of life scale. *Stroke* 1999;30(7):1362-9.
37. Thornton M, Travis SS. Analysis of the reliability of the modified caregiver strain index. *J Gerontol B, Psychol Sci Soc Sci* 2003;58(2):S127-32.
38. Craig LE, Wu O, Bernhardt J, et al. Approaches to economic evaluations of stroke rehabilitation. *Int J Stroke* 2014;9(1):88-100.
39. Browne RH. On the use of a pilot sample for sample size determination. *Stat Med* 1995;14(17):1933-4.

For peer review only - http://bmjopen.bmj.com/site/about/guidelines.xhtml

40. Bruno A, Akinwuntan AE, Lin C, et al. Simplified Modified Rankin Scale Questionnaire: Reproducibility Over the Telephone and Validation With Quality of Life. *Stroke* 2011; 42(8)2276-9.
41. Hislop J, Adewuyi TE, Vale LD, et al. Methods for specifying the target difference in a randomised controlled trial: the Difference ELicitation in TriAls (DELTA) systematic review. *PLoS Med* 2014;11(5):e1001645.
42. McKenna S, Jones F, Glenfield P, et al. Bridges self-management program for people with stroke in the community: A feasibility randomized controlled trial. *Int J Sroke* 2015;10(5):697-704.
43. Wade DT. Outcome measures for clinical rehabilitation trials: impairment, function, quality of life, or value? *Am J Phys Med Rehabil* 2003;82(10 Suppl):S26-31.
44. Rehabilitation Institute of Chicago. Rehabilitation Measures Database - Stroke Impact Scale [Available from: http://www.rehabmeasures.org/Lists/RehabMeasures/DispForm.aspx?ID=934 accessed 21 06 2017.
45. Saywell N, Vandal AC, Brown P, et al. Telerehabilitation to improve outcomes for people with stroke: study protocol for a randomised controlled trial. *Trials* 2012;13(1):233.

For peer review only

For peer review only - http://bmjopen.bmj.com/site/about/guidelines.xhtml

Figure 1a – Recruitment and randomisation by cohort

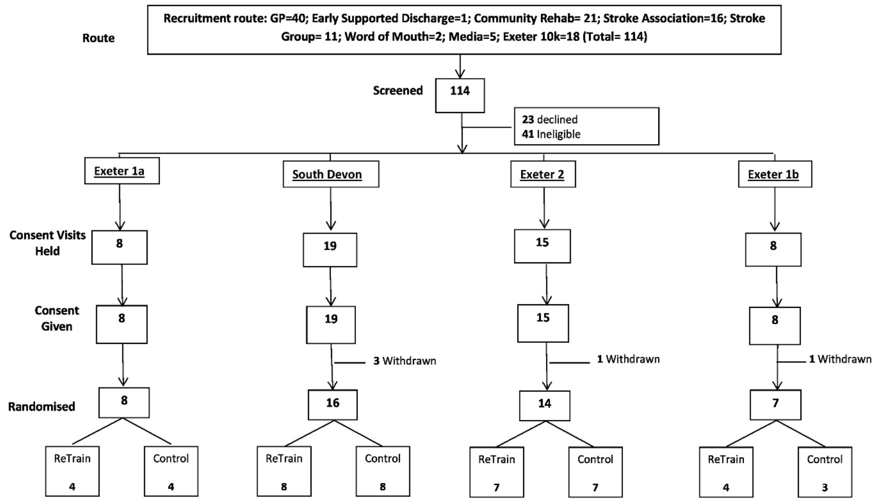

Figure 1a - Recruitment and randomisation by cohort

297x209mm (300 x 300 DPI)

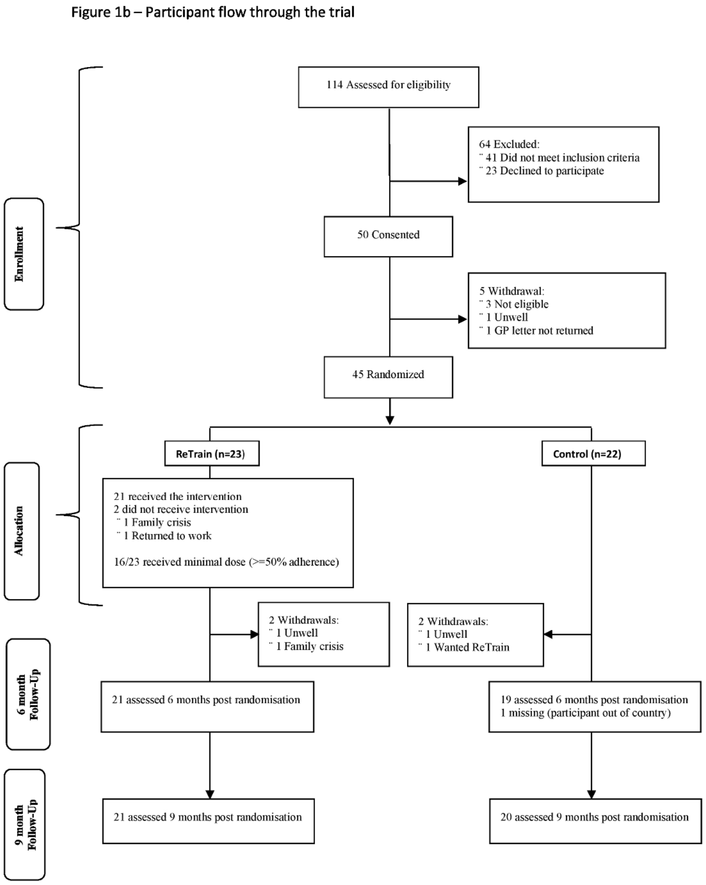

Figure 1b - Participant flow through the trial

209x297mm (300 x 300 DPI)

Table 2 | CONSORT checklist of information to include when reporting a pilot trial

Section/topic and item No	Standard checklist item	Extension for pilot trials	Page No where item is reported
**Title and abstract**			
1a	Identification as a randomised trial in the title	Identification as a pilot or feasibility randomised trial in the title	1
1b	Structured summary of trial design, methods, results, and conclusions (for specific guidance see CONSORT for abstracts)	Structured summary of pilot trial design, methods, results, and conclusions (for specific guidance see CONSORT abstract extension for pilot trials)	2
**Introduction**			
Background and objectives:			
2a	Scientific background and explanation of rationale	Scientific background and explanation of rationale for future definitive trial, and reasons for randomised pilot trial	4
2b	Specific objectives or hypotheses	Specific objectives or research questions for pilot trial	4-5
**Methods**			
Trial design:			
3a	Description of trial design (such as parallel, factorial) including allocation ratio	Description of pilot trial design (such as parallel, factorial) including allocation ratio	5
3b	Important changes to methods after trial commencement (such as eligibility criteria), with reasons	Important changes to methods after pilot trial commencement (such as eligibility criteria), with reasons	N/A
Participants:			
4a	Eligibility criteria for participants		5
4b	Settings and locations where the data were collected		5-6
4c		How participants were identified and consented	5
Interventions:			
5	The interventions for each group with sufficient details to allow replication, including how and when they were actually administered		5-6
Outcomes:			
6a	Completely defined prespecified primary and secondary outcome measures, including how and when they were assessed	Completely defined prespecified assessments or measurements to address each pilot trial objective specified in 2b, including how and when they were assessed	6
6b	Any changes to trial outcomes after the trial commenced, with reasons	Any changes to pilot trial assessments or measurements after the pilot trial commenced, with reasons	N/A
6c		If applicable, prespecified criteria used to judge whether, or how, to proceed with future definitive trial	N/A
Sample size:			
7a	How sample size was determined	Rationale for numbers in the pilot trial	6-7
7b	When applicable, explanation of any interim analyses and stopping guidelines		N/A
Randomisation:			
Sequence generation:			
8a	Method used to generate the random allocation sequence		7
8b	Type of randomisation; details of any restriction (such as blocking and block size)	Type of randomisation(s); details of any restriction (such as blocking and block size)	7
Allocation concealment mechanism:			
9	Mechanism used to implement the random allocation sequence (such as sequentially numbered containers), describing any steps taken to conceal the sequence until interventions were assigned		7
Implementation:			
10	Who generated the random allocation sequence, enrolled participants, and assigned participants to interventions		7
Blinding:			
11a	If done, who was blinded after assignment to interventions (eg, participants, care providers, those assessing outcomes) and how		7
11b	If relevant, description of the similarity of interventions		N/A
Analytical methods:			
12a	Statistical methods used to compare groups for primary and secondary outcomes	Methods used to address each pilot trial objective whether qualitative or quantitative	7
12b	Methods for additional analyses, such as subgroup analyses and adjusted analyses	Not applicable	N/A

For peer review only - http://bmjopen.bmj.com/site/about/guidelines.xhtml

RESEARCH METHODS AND REPORTING

Table 2 | CONSORT checklist of information to include when reporting a pilot trial

Section/topic and item No	Standard checklist item	Extension for pilot trials	Page No where item is reported
**Results**			
Participant flow (a diagram is strongly recommended):			
13a	For each group, the numbers of participants who were randomly assigned, received intended treatment, and were analysed for the primary outcome	For each group, the numbers of participants who were approached and/or assessed for eligibility, randomly assigned, received intended treatment, and were assessed for each objective	Figure 1a & 1b & P.8
13b	For each group, losses and exclusions after randomisation, together with reasons		Figure 1a & 1b & p.10
Recruitment:			
14a	Dates defining the periods of recruitment and follow-up		8
14b	Why the trial ended or was stopped	Why the pilot trial ended or was stopped	N/A
Baseline data:			
15	A table showing baseline demographic and clinical characteristics for each group		8 - 10
Numbers analysed:			
16	For each group, number of participants (denominator) included in each analysis and whether the analysis was by original assigned groups	For each objective, number of participants (denominator) included in each analysis. If relevant, these numbers should be by randomised group	Figure 1b
Outcomes and estimation:			
17a	For each primary and secondary outcome, results for each group, and the estimated effect size and its precision (such as 95% confidence interval)	For each objective, results including expressions of uncertainty (such as 95% confidence interval) for any estimates. If relevant, these results should be by randomised group	N/A
17b	For binary outcomes, presentation of both absolute and relative effect sizes is recommended	Not applicable	N/A
Ancillary analyses:			
18	Results of any other analyses performed, including subgroup analyses and adjusted analyses, distinguishing prespecified from exploratory	Results of any other analyses performed that could be used to inform the future definitive trial	10 - 15
Harms:			
19	All important harms or unintended effects in each group (for specific guidance see CONSORT for harms)		16
19a		If relevant, other important unintended consequences	N/A
**Discussion**			
Limitations:			
20	Trial limitations, addressing sources of potential bias, imprecision, and, if relevant, multiplicity of analyses	Pilot trial limitations, addressing sources of potential bias and remaining uncertainty about feasibility	17 - 18
Generalisability:			
21	Generalisability (external validity, applicability) of the trial findings	Generalisability (applicability) of pilot trial methods and findings to future definitive trial and other studies	18
Interpretation:			
22	Interpretation consistent with results, balancing benefits and harms, and considering other relevant evidence	Interpretation consistent with pilot trial objectives and findings, balancing potential benefits and harms, and considering other relevant evidence	18 - 19
22a		Implications for progression from pilot to future definitive trial, including any proposed amendments	19
**Other information**			
Registration:			
23	Registration number and name of trial registry	Registration number for pilot trial and name of trial registry	2, 19
Protocol:			
24	Where the full trial protocol can be accessed, if available	Where the pilot trial protocol can be accessed, if available	19
Funding:			
25	Sources of funding and other support (such as supply of drugs), role of funders		2, 19
26		Ethical approval or approval by research review committee, confirmed with reference number	19

For peer review only - http://bmjopen.bmj.com/site/about/guidelines.xhtml

Community-based Rehabilitation Training after stroke: Results of a pilot randomised controlled trial (ReTrain) investigating acceptability and feasibility

Journal:	*BMJ Open*
Manuscript ID	bmjopen-2017-018409.R1
Article Type:	Research
Date Submitted by the Author:	24-Aug-2017
Complete List of Authors:	Dean, Sarah; PenCLAHRC University of Exeter Medical School,<br>Poltawski, Leon; University of Exeter Medical School<br>Forster, Anne; University of Leeds, Academic Unit of Elderly Care and Rehabilitat<br>Taylor, Rod; University of Exeter, Peninsula Medical School<br>Spencer, Anne<br>James, Martin; South West NHS Trust<br>Allison, Rhoda; Torbay and Southern Devon Health and Care NHS Trust<br>Stevens, Shirley; University of Exeter Medical School<br>Norris, Meriel; Brunel University London<br>Shepherd, Anthony; University of Portsmouth, Sport and Exercise Science<br>Landa, P; University of Exeter, Medical School<br>Pulsford, Richard; University of Exeter, Sports and Health Science<br>Hollands, Laura; University of Exeter Medical School<br>Calitri, Raff; University of Exeter Medical School
**Primary Subject Heading**:	Rehabilitation medicine
Secondary Subject Heading:	Cardiovascular medicine, Health services research
Keywords:	STROKE MEDICINE, REHABILITATION MEDICINE, Clinical trials < THERAPEUTICS

Title Community-based Rehabilitation Training after stroke: Results of a pilot randomised controlled trial (ReTrain) investigating acceptability and feasibility

Sarah G Dean[1], Leon Poltawski[1], Anne Forster[2], Rod S Taylor[1], Anne Spencer[1], Martin James[1,3], Rhoda Allison[4], Shirley Stevens[1], Meriel Norris[5], Anthony I Shepherd[6], Paolo Landa[1], Richard M Pulsford[7], Laura Hollands[1], Raff Calitri[1].

[1]University of Exeter Medical School, Exeter, EX1 2LU, UK.

[2]Academic Unit of Elderly Care and Rehabilitation, University of Leeds, Bradford, BD9 6RJ, UK.

[3]Royal Devon & Exeter Hospital, Exeter, EX2 5DW, UK.

[4]Torbay and South Devon NHS Foundation Trust, Torquay, TQ2 7TD, UK.

[5]Department of Clinical Sciences, Brunel University London, Middlesex UB8 3PH, UK.

[6] Department of Sport and Exercise Science, University of Portsmouth, Portsmouth, PO1 2ER.

[7]Sports & Health Sciences, University of Exeter, Exeter, EX1 2LU, UK.

Corresponding author

Associate Professor Sarah Dean,

Psychology Applied to Rehabilitation and Health,

University of Exeter Medical School,

College House

St Luke's Campus,

Exeter,

EX1 2LU,

Email: s.dean@exeter.ac.uk

01392 722984

Word count 4484 exc tables

For peer review only

Key words: Stroke, Rehabilitation Medicine, Clinical Trials.

Abstract

Objectives: To assess acceptability and feasibility of trial processes and the ReTrain intervention including an assessment of intervention fidelity. **Design:** A two-group, assessor-blinded, randomised controlled trial with parallel mixed methods process and economic evaluations**. Setting:** Community settings across two sites in Devon. **Participants**: Eligible participants were: 18 years old or over, with a diagnosis of stroke and with self-reported mobility issues, no contraindications to physical activity, discharged from National Health Service (NHS) or any other formal rehabilitation programme at least 1 month prior, willing to be randomised to either control or ReTrain and attend the training venue, possessing cognitive capacity and communication ability sufficient to participate. Participants were individually randomised (1:1) via a computer generated randomisation sequence minimised for time since stroke and level of functional disability. Only outcome assessors independent of the research team were blinded to group allocation. **Interventions:** ReTrain comprised (1) an introductory one-to-one session; (2) ten, twice weekly group classes with up to two trainers and eight clients; (3) a closing one-to-one session, followed by three drop-in sessions over the subsequent three months. Participants received a bespoke home-based training programme. All participants received treatment as usual. The control group received an exercise after stroke advice booklet. **Outcome measures:** Candidate primary outcomes included functional mobility and physical activity. **Results:** Forty-five participants were randomised (ReTrain=23; Control=22); data were available from 40 participants at six months follow-up (ReTrain=21; Control=19) and 41 at nine months follow-up (ReTrain=21; Control=20). We demonstrated ability to recruit and retain participants. Participants were not burdened by the requirements of the study. We were able to calculate sample estimates for candidate primary outcomes and test procedures for process and health economic evaluations. **Conclusions:** All objectives were fulfilled and indicated that a definitive trial of ReTrain is feasible and acceptable.

Registration: ClinicalTrials.gov: trial number NCT02429180.

Funding: The Stroke Association TSA 2014-13

For peer review only - http://bmjopen.bmj.com/site/about/guidelines.xhtml

Strengths and limitations of this study

- A community-based exercise intervention after stroke developed with service users
- A pilot randomised trial developed following MRC guidelines on complex interventions
- Mixed-method approach for answering feasibility and acceptability objectives
- Objective physical activity capture via accelerometry but subjective self-report outcomes for functional mobility and psycho-social measurements

For peer review only

For peer review only - http://bmjopen.bmj.com/site/about/guidelines.xhtml

Introduction

Five years after initial stroke, one in three individuals have residual physical impairment[1], equating to over 300,000 individuals in the United Kingdom (UK) living with disability from stroke[2]. Provision of stroke rehabilitation is typically front loaded, with resources focussed on in-patient care and early supported discharge. Support tapers off after a few months[3] with many individuals reporting unmet long-term needs[4].

The National Clinical Guideline for Stroke advise for secondary prevention that stroke survivors engage in 150 minutes of physical activity a week, in bouts of 10 minutes or more, starting light and developing across time to moderate levels of intensity[5]. However, many stroke survivors do not meet these recommendations[6 7] due to combinations of personal (e.g., physical or psychological impairments) and environmental factors (e.g., lack of programmes and facilities). To address this problem, community-based programmes are promoted[8-10]. These tend to focus on cardiovascular fitness with less emphasis on functional improvements or on promoting on-going exercise self-management. National stroke guidelines[5] identify the importance of interventions for functional improvement[11] and self-management[12] but evidence is lacking regarding these types of intervention[13].

Action for Rehabilitation following Neurological Injury (ARNI) is an approach aimed at improving function and facilitating self-management[14] and has a detailed self-help book. The ARNI approach embodies a set of principles (e.g. instilling a commitment to regular exercise) and techniques tailored to individual need. The ARNI Institute trains registered exercise professionals to deliver key ARNI techniques. Clinical Commissioning Groups (CCGs), charitable, and local authorities have started to provide community-based ARNI training for stroke survivors, which has been positively received by participants, carers and practitioners[15], however there is currently no randomised controlled trial (RCT) evidence for evaluating its impact on stroke outcomes or its cost-effectiveness.

Background and objectives

Using the Medical Research Council's framework for the development and evaluation of complex interventions[16] and considerable Patient and Public Involvement we have designed a testable programme called Rehabilitation Training (ReTrain)[17-20]. ReTrain is a community-based, manualised group programme combining ARNI principles and key techniques with best practice guidelines for stroke[9 17]. The overall aim of our pilot RCT was to inform the design and delivery of a definitive RCT. Our objectives were to: 1) assess feasibility and acceptability of recruitment, randomisation, allocation concealment and outcome assessment blinding; 2) determine retention rates; 3) check ReTrain's acceptability and feasibility for participants, and refine the Trainer Manual; 4) test candidate outcome measures, assess their burden, levels of completion, and estimate outcome variance (to inform definitive trial sample size); 5) perform process evaluation including intervention

For peer review only - http://bmjopen.bmj.com/site/about/guidelines.xhtml

fidelity assessment; 6) calculate ReTrain costs and assess feasibility of collecting health and social service resource use.

Methods

A brief methods overview is provided in accordance with guidance for reporting pilot trials[21]; further details are available in the published protocol[22]. Ethics review was conducted by National Research Ethics Service Committee South West Cornwall & Plymouth (REC Ref: 15/SW/04).

Trial design

ReTrain was a two-group, assessor-blinded, randomised controlled external pilot trial with parallel mixed methods process and economic evaluations. Eligible participants were individually randomised 1:1 to intervention (ReTrain) or control (exercise advice booklet[23]).

Participants

Inclusion criteria were: i) diagnosis of stroke; ii) any time since stroke but at least 1 month since discharge from NHS physical rehabilitation services; iii) able to walk independently indoors with or without mobility aids, but with self-reported difficulty with stairs, slopes or uneven surfaces; iv) willingness to be randomised and attend the training venue; v) cognitive capacity and communication ability sufficient to participate.

Exclusion criteria were: less than 18 years old, currently (or within one month of) receiving ARNI training or have contraindications to moderate to vigorous physical activity (adapted from American College of Sports Medicine guidelines[24]). Participants were recruited from two CCGs. Participants were identified by: (1) clinicians in NHS primary care, hospital and community stroke services; (2) contacts in the local Clinical Research Network and Clinical Research Facility; (3) promotion via local stroke support networks (e.g. Stroke Association); (4) word of mouth, study flyers and adverts.

Intervention

ReTrain aims to: (1) enhance function through task-related practice, teaching compensatory techniques, and providing targeted strength training (cardiovascular fitness gains also occur through these activities); (2) develop self-management skills for on-going rehabilitation; (3) deliver personalised training using negotiated goals and (4) instil a commitment to regular exercise for health improvement and longer-term maintenance. ReTrain facilitates safe and efficient practice of walking in varied terrains, kerbs, cambers and in crowds, turning and moving quickly, climbing steps and stairs without rails, getting to and from the floor without furniture or other aids, and moving without mobility aids or while carrying loads. Training is based on a manual and led by personal trainers on the UK Register of Exercise Professionals (level 3 or above) who are ARNI-trained and accredited and have had additional training in the delivery of ReTrain. There was a maximum ratio of one trainer to four stroke survivors.

For peer review only - http://bmjopen.bmj.com/site/about/guidelines.xhtml

ReTrain was delivered in a community setting (one gym, two church halls, one community centre) with twice weekly two hour sessions over three months, comprising: an introductory one-to-one session (home visit); ten, twice-weekly group classes with up to two trainers and eight clients (training venue); a closing one-to-one session (home visit); followed by three (one per month) drop-in sessions. Participants completed bespoke home-based training (homework) throughout.

Control

All participants received treatment as usual. This ranged from zero treatment to engagement with any health service(s). We requested that all trial participants did not participate in additional physical rehabilitation (either NHS or private) but we could not prevent them from doing so. We did not monitor control group participation in any treatments during the trial but did record health service use at the end of the trial for all participants. The control group also received an advice booklet about exercise after stroke[23].

Outcomes

Feasibility, acceptability and process outcomes: numbers and details of those approached; recruitment and retention figures. ***Acceptability of randomisation, outcome measurement burden, and the intervention:*** completion of questionnaires and objective assessments; interviews with ten intervention and ten control group members, and the trainers. ***Safety:*** Adverse events[25] identified via trainer and ReTrain participants (during the programme) and participant reports (all participants during 6 and 9-month assessments). ***Intervention fidelity:*** attendance registers, accelerometry, exercise 'homework' diaries, trainer completed session checklists and video analysis of (early, middle and late programme) training sessions.

We tested a range of candidate primary and secondary outcome measures. ***Primary Outcomes***: Rivermead Mobility Index[26 27]; Timed Up and Go Test[28]; modified Patient-Specific Functional Scale[29]; 7-day objective physical activity levels using wrist-worn accelerometry (GENEActiv, Activinsights, Kimbolton, Cambridge UK) and a physical activity diary. ***Secondary Outcomes***: Stroke Self-efficacy Questionnaire[30]; Fatigue Assessment Scale[31 32]; exercise beliefs and exercise self-efficacy questionnaires[33]; SF12[34]; EQ-5D-5L[35]; Stroke Quality of Life (QoL) questionnaires[36]; Carer Burden Index[37]; and Health and Social Service use through a Service Receipt Inventory[38].

Physical outcome baseline assessments (completed by research team) and follow-up assessments (at 6 and 9-months, completed by blinded assessor) were conducted in the participant's home. Researchers visited participants to fit the accelerometer, drop off questionnaires and diary one week prior to blind assessor visits. Assessors administered primary outcome physical measures and collected accelerometers, questionnaires and diaries.

For peer review only - http://bmjopen.bmj.com/site/about/guidelines.xhtml

Sample size

We required 48 participants (24 per group) as (a) 30 complete data sets are recommended for pilot studies to estimate outcome variance[39] and (b) we wanted to investigate variations in context by running the intervention three times (*i.e.* 3 x 8 patients). This number also allowed estimation of a predicted attrition rate of 20% with a precision of ± 5% with 95% certainty.

Randomisation and blinding

The random sequence was computer generated with minimisation for time since stroke (≤ 3 months versus > 3 months) and level of functional disability (modified Rankin Scale (mRS)[40] score ≤ 2 versus > 2). Allocation concealment was ensured by using a password protected validated web-based remote randomisation service supported by the Peninsula Clinical Trials Unit (PenCTU). The Trial Manager requested randomisation only after a cohort of participants had been consented.

Participants, trainers providing the intervention, and researchers conducting the process and economic evaluations could not be blinded to allocation. However, outcomes were assessed by independent researchers (not based at research centre) who were blinded to group allocation. Participants were reminded not to reveal their allocation to assessors but any un-blinding was recorded; after assessments assessors were asked to guess participant allocation.

Data Analysis

Analysis was primarily descriptive with participant flow summarised and estimates of screening, recruitment and attrition reported. Means and standard deviations for all outcomes are reported at baseline, 6 and 9-months follow-up for each group.

Intervention fidelity was assessed using mixed methods: qualitative video analysis comparing the Trainer Manual standard versus observed technique (two researchers independently assessed videos) combined with interview data and summary scores from trainer completed session checklists. Qualitative data were manually analysed descriptively and with content analysis for trial processes; additional thematic analysis was used for interview data. One person (MN) led the qualitative analysis but this was then discussed (MN & SD), checked (RC) and agreed (MN, SD, LP, RC).

We used a micro-costing approach to calculate costs associated with ReTrain: staff time (trainers, administrator, facilitators), venue hire, training equipment (annualised over time), course materials, consumables, travel costs (participants, trainers and facilitators). The costs of the intervention were estimated as a cost per programme and a cost per participant. The estimated costs of the intervention per participant were based on the number of participants enrolled on the programme. The base case scenario assumed the average

For peer review only - http://bmjopen.bmj.com/site/about/guidelines.xhtml

number of participants per programme across all cohorts. Sensitivity analyses were conducted using the minimum and maximum number of participants enrolled for the programme and the quantity of programme materials that were wasted. We analysed the relative benefits of calculating health related QoL using SF-6D (developed from the SF-12) over the QALY calculated (using EQ-5D 5L) from the baseline measures.

Sample size estimates for a definitive trial were calculated for candidate primary outcomes using the standard deviation observed in this pilot population and published minimal clinical important difference (MCID) at 90% power and 5% alpha, and assuming 20% attrition. Where no published MCID could be sourced, we assumed a small to moderate effect size of 0.4 of a standard deviation[41]. The trial statistician undertook calculations using the 'samspi' command in STATA v14.2

Results

Recruitment took place from June 2015 to January 2016. The intervention ran in four cohorts, participant flows are shown for each (Figure 1) and for the trial overall (Figure 2). Initial recruitment was slow so to prevent late running of the trial we split the first cohort. Six-month follow-up outcome assessments took place January to July 2016 and 9-month follow-up April to October 2016.

Objective 1: *Assess the feasibility and acceptability of recruitment, randomisation, allocation concealment and processes for outcome assessment and blinding*

We screened 115 individuals to recruit 50 participants (Figure 1) in 8 months (2 months ahead of schedule). Of these, 45 (90%) were randomised (Figure 1 and 2). Five individuals withdrew prior to randomisation due to ill health or the time lag between agreeing to take part and a cohort being ready to randomise. Table 1 shows baseline characteristics of those randomised, indicating a balance of characteristics across trial arms.

Table 1 Baseline participant demographics

	ReTrain (N= 23)	Control (N=22)
**Gender, n**		
Male (%)	16 (70%)	14 (67%)
**Age (years): mean (SD)**	70 (12)	71 (10)
**Age Category (N=45): n (%)**		
<45	1 (4%)	0 (%)
46-50	0 (0%)	1 (5%)
51-60	3 (13%)	2 (9%)
61-70	10 (43%)	6 (27%)
71-80	5 (22 %)	8 (36%)
81-90	2 (9%)	5 (23%)
90+	2 (9%)	0 (0%)
**Time Since Stroke (no. months):**		

For peer review only - http://bmjopen.bmj.com/site/about/guidelines.xhtml

< 12	3 (13%)	3 (14%)
12-24	4 (17%)	4 (18%)
25-48	5 (22%)	5 (23%)
49-72	2 (9%)	5 (23%)
73-96	4 (17%)	2 (9%)
97+	5 (22%)	3 (14%)
**Time Since Stroke Minimisation Categories (months):n, (%)**		
<=3 months	1 (4%)	0 (0%)
>3 months	22 (96%)	22 (100%)
**Type of Stroke, n (%)**		
Haemorrhagic	3 (13%)	1 (5%)
Ischaemic	15 (65%)	15 (68%)
Both	0 (0%)	1 (5%)
Missing	5 (22%)	5 (23%)
**Stroke Rehabilitation (weeks):**		
n,<br>Average no. weeks (SD)	21,<br>8 (9)	21,<br>14 (19)
Median no. weeks	6	12
Range	0-32	0-88
Unknown length rehab: n	2	1
**Functional Disability (Simplified Modified Rankin Scale score- sMRS): n (%)**		
0	1 (4%)	0 (0%)
1	2 (9%)	1 (5%)
2	4 (17%)	9 (41%)
3	16 (70%)	12(55%)
**sMRS minimisation categories: n(%)**		
<=2	7 (30%)	10 (45%)
>2	16 (70%)	12 (55%)
**Co-morbidities^, n (%)**		
Hypertension	18 (78%)	18 (82%)
Type 2 Diabetes Mellitus	4 (17%)	4 (18%)
Depression	8 (35%)	5 (23%)
Chronic Kidney Disease	2 (9%)	1 (4%)
Asthma / COPD	4 (17%)	3 (14%)
Other	5 (22%)	3 (14%)
**Medications^, n (%)**		
Diuretics	3 (13%)	1 (5%)
Anticoagulants	8 (35%)	10 (45%)
Antiplatelet	15 (65%)	12 (55%)
Antihypertensives<br>Calcium Channel Blockers<br>ACE inhibitors<br>Other	 6 (26%)<br>13 (57%)<br>9 (39%)	 14 (64%)<br>8 (36%)<br>7 (32%)
Statins	18 (78%)	19 (86%)
Anti-depressants	8 (35%)	5 (23%)
Diabetes medication	4 (17%)	4 (18%)

For peer review only - http://bmjopen.bmj.com/site/about/guidelines.xhtml

Chronic pain medication	12 (52%)	8 (36%)
Other	5 (22%)	3 (14%)
**Employment Status, n (%)**		
Employed (and working)	2 (9%)	1 (5%)
Retired	18 (78%)	15 (68%)
Semi-retired	1 (4%)	0 (0%)
Unemployed	2 (9%)	5 (27%)
**Pre-stroke Exercise History, n**		
Exerciser (%)	10 (43%)	8 (36%)
**Mini Mental State Exam:**		
n,<br>Mean (SD)	22*,<br>27.5 (2.54)	22,<br>27.9 (3.01)
Median	28	29
Range	19– 30**	19-30**

^ Participants may have more than one co-morbidity / medication.
*1 participant with severe aphasia had difficulties completing the MMSE. The participant could understand and follow instructions and was considered cognitively able to participate in the trial.
**Higher scores indicate better cognitive function. Participants range from no to moderate degree of cognitive impairment.

Blinding of outcome assessors was considered successful as only 2/41 (5%) participants revealed their allocations after completion of outcome measures, both were intervention participants. Different assessors were used for subsequent assessments therefore risk of bias was minimised.

Objective 2: *Acquire retention rates and outcome variance*

Forty out of 45 (88%, 95% CI: 76% to 96%) completed 6-month and 9-month follow-ups. Despite fewer people being randomised than expected, high retention preserved the number of datasets needed to perform our sample size estimates (Table 2).

Table 2 Sample estimates for potential candidate primary outcomes from ReTrain pilot RCT

Primary Outcome Measure	Sample Size Estimates[a]	Minimal Clinically Important Difference (MCID)	Observed SD range	Effect size (MCID/SD)
Rivermead Mobility Index	36 – 44	3.0[b]	2.33 – 2.66	1.13 – 1.29
Timed Up and Go	1,438 – 2,673	1.2 – 3.4[c]	15.69 – 21.39	0.06 – 0.22
Modified Patient Specific Functional Scale	16 – 200	1.0 – 3.0[c]	1.58 – 1.94	0.52 – 1.7

For peer review only - http://bmjopen.bmj.com/site/about/guidelines.xhtml

Physical Activity (Accelerometer)	350 - 1458	Not available	Not applicable	0.2 – 0.45[d]

[a]Figures represent overall (two group) sample size estimates required for a definitive trial. Sample sizes estimated for 1:1 allocation at 90% power and 5% alpha and assuming 20% attrition. Calculations are conservative showing range from best case scenario (largest MCID and smallest SD) to worst case scenario (smallest MCID and largest SD) of SDs observed in this trial and published MCIDs where available.

[b]MCID available from stroke research for the Rivermead Mobility Index (http://www.strokengine.ca/psycho/rmi_psycho/).

[c]MCIDs identified from other disease groups used as proxies as no published stroke MCIDs[42,43].

[d]There are no MCID data available for PA (accelerometry) in stroke (or any other cardio vascular disease) we therefore applied sample size calculations undertaken for a relevant ongoing HTA NIHR trial which estimated n= 562 (effect size 0.3) or n= 413 (effect size 0.35) (http://www.isrctn.com/ISRCTN15644451).

Objective 3: *Acceptability and feasibility for participants and to complete the Trainer manual*

Eleven themes from 20 qualitative interviews summarise participants' views, Table 3 provides illustrative quotes.

For peer review only

Table 3 Participant quotes from qualitative interviews

**Acceptability**
"It is ten weeks, you do it twice a week. Personally for the first say three or four weeks, I'd think well this is getting me nowhere, but then you think that you notice things, things are improving and at the end of ten weeks you want to go for twenty weeks (4:119-125)
"I'd tell them [another stroke survivor] to go ahead and do it and to take it step by step and not to worry about it. Because you are treated with great respect, it was wonderful and they were. I'll never be able to speak highly enough of them." (25:388-390)
Intervention approach
"It opened my eyes to what can be done you know. How can I put it? It wasn't as if I believed that I couldn't do something it was being pointed in the right direction…heh I can do it…Great you've done it, you did it and you do it again. Yeah it was great" (4:358-361). "It wasn't easy at first, but I used to manage it" (5:246)
"It was the way they addressed how you do your exercises. What it is doing to you and all the rest of it. Now to me that was absolutely important, because it made sense of why you are doing all this pumping up and down, and if you can't do that, do this." (22:252-255)
"It was you felt as if you were a human being with them. You know and you were treated with respect…and although you couldn't do things and you felt a bit of an idiot, they never let you feel like that" (25:567-572).
"It's a bit like playing scales…it's not creative but as I gradually realise it, it could potentially be creative…doing something that I had been doing without thinking before and now couldn't. …Now and again I walk without my stick without realising it, that's creative I think." (6:354-392)
Impact of programme - psychologically
"I suppose it is attitude of mind as much as anything. I mean I felt I'd gone through that stage of training and that I was going to get better. It built my spirit up…I felt as if it, well it was worth the three months you know and at the end of the day I hope I'm going to get back to something like normal" (16:358-365)
"It really helped me mentally, you know I thought right I can do this because before I was going into my shell, thinking I can't do this and I can't do that. Oh I am not going out. Then I went on that [ReTrain] and it gave me an element of confidence." (43:562-565).
Impact of programme – physically
"you started to notice they are actually starting to fall into place. I don't remember doing that last time. But I am doing it now great get on with it I am doing it faster now" (4:189-190)
"I know if I went down which I did one day in the hall in the early stages of coming back home and I did manage to get up and walk upstairs…but I wouldn't have been able to do that had I not had that [training]" (16:475-477)
Homework adherence
"trainers were always on about doings exercises at home…I could never pin him down to how long that should be for though" (6; line 624).
Programme technique adherence
"I think that was the big thing you saw the benefits after the second, well the first or second session we had. 'Oh we can do

a) Study Information

Participants considered information received as adequate. Five noted that information was limited, but most were unconcerned. Two added that too much information may have been detrimental to recruitment. Four others were satisfied with the information they received.

b) Outcome Measure Burden

Participants found the assessment process acceptable. Fifteen indicated no burden. Three participants indicated that they needed help from their carers to complete questionnaires,

For peer review only - http://bmjopen.bmj.com/site/about/guidelines.xhtml

particularly recalling and reporting health resource use, placing a time burden on their carer.

c) Venue

Half of the ReTrain participants were very positive about the training venues. Important features were: space, provision of fluids (water, tea), easy availability of parking. For some the travelling distance was a concern; two noted their venue (a gym) was very noisy, insufficiently heated and the session time was too early. Some noted the small amount of equipment as an advantage (it aided transfer of exercises to their home) whereas others felt the equipment was not sufficiently specialist.

d) Adherence to ReTrain (see also Objective 5)

All ten ReTrain interviewees reported training in the five core (ARNI) techniques. Homework was discussed by all but lacked specificity, only two had clear homework examples that were effectively incorporated into their training. Although goal setting was a core element, only four specifically identified how their goals were linked into their overall programme. Three participants reported not attending drop-in sessions due to lack of information. Of three who attended two suggested the drop-ins repeated previous sessions.

e) Group dynamics

Group working was positively regarded and seen as integral to programme effectiveness. There were exceptions, one participant did not find ‘performing’ in public a positive experience. Likewise some suggested that groups reduced training intensity relative to one-to-one training.

f) Co-morbidities

Participants identified several co-morbidities e.g.: knee replacements, cancer, angina, diabetes, amputation and depression. These had potential to impact on both the training and research participation but for most any concerns were accommodated by trainers. However, in one case some uncomfortable discussions occurred before an appropriate balance of perceived capability and training challenges was reached. Three participants with visual deficits, dyslexia and dysgraphia mentioned difficulties completing the research documents.

g) Carer Health

Two ReTrain participants commented on how commitment to the programme impacted on their partner’s health: one stopped attending sessions because the time away resulted in excessive strain on his wife; another expressed similar concern but did not stop attending.

h) Trainer Manual

For peer review only - http://bmjopen.bmj.com/site/about/guidelines.xhtml

We refined the Trainer Manual throughout the study. Issues raised during interviews guided revisions including greater emphasis and clarification about use of goal setting, drop-ins, homework diaries, and managing participants with co-morbidities.

Objective 4: *Assess outcome completion and burden*

We collected baseline (n=41), 6-month (n=40) and 9-month (n=41) follow-up data on the majority of participants (Figure 2). Accelerometry wear time (24 hours for 7 days) was high, most having 6 or more valid days (≥ 16hrs per day, including ≥ 1 weekend day). Only two participants at baseline, one at 6-months and three at 9-months did not achieve 4 valid days of wear time. There was very little missing data. For three primary outcome measures there was only one participant with missing data at any given assessment time-point. For secondary outcomes there was either no missing data or only one to two participants with missing data at each time-point, apart from the exercise diary (between two and four participants with missing data at each time-point) and the Service Receipt Inventory (between three and seven participants with missing data). There were eight participants without accelerometer data at 9-month assessment owing to hardware (device) and software (data extraction method) malfunctions.

Objective 5: *Perform process evaluation with an assessment of intervention fidelity*

We implemented a comprehensive video recording schedule (over 200 recordings) to capture participant and trainer adherence to key ARNI techniques. Both trainers and participants demonstrated high adherence. Modifications to techniques (to accommodate participant co-morbidities) were captured and informed Trainer Manual development.

We combined metrics from attendance registers and homework records to generate a ‘dose’/adherence score, categorising individuals into low (< 50%), medium (50% - 75%) and high (>75%) adherence categories. Of 23 ReTrain participants two did not receive the intervention (one returned to work; one withdrew from study), five had low adherence, five medium adherence, and eleven high adherence. These latter 16 (70%) were considered to have received sufficient ‘dose’ of ReTrain.

Trainers varied in their completion of session checklists: pre-exercise and end-of-session components were less consistently reported compared to ARNI techniques but overall there was good adherence to programme delivery.

Objective 6: *Calculate the cost of intervention delivery and feasibility of collecting health and social service resource use.*

ReTrain costs were generated for each cohort, accounting for different programme sizes (four or eight participants) and venues. Costs per participant ranged from £615 to £972. The total per participant cost for ReTrain (assuming 24 participants) was £777. We conducted medical notes review on 35/41 participants and compared this ‘gold standard’ with self-reported health resource use. Participants reported using fewer resources compared to case

For peer review only

For peer review only - http://bmjopen.bmj.com/site/about/guidelines.xhtml

notes review. Data from medical notes informed the cost-utility and effectiveness frameworks for use in a definitive trial.

Descriptive analysis of participant outcomes

Primary and secondary outcome data are summarised in Table 4a and 4b respectively.

For peer review only

For peer review only - http://bmjopen.bmj.com/site/about/guidelines.xhtml

Table 4a: Number, means and standard deviations as a function of trial arm and measurement time point for candidate primary outcome measures in the ReTrain pilot trial

	Data collection time point					
	Baseline		6 month^		9 month^	
**Measures:** n, Mean (SD)	**ReTrain (N=23)**	**Control (N=22)**	**ReTrain (N=21)**	**Control (N=20)**	**ReTrain (N=21)**	**Control (N=20)**
Rivermead Mobility Index	23, 11.41 (3.05)	22, 11.68 (2.23)	21, 12.14 (2.73)	19, 12.47 (1.87)	21, 12.24 (3.27)	20, 12.65 (1.81)
Modified Patient Specific Functional Scale	22, 2.95 (1.85)	22, 2.55 (1.23)	21, 3.47 (2.12)	19, 3.56 (1.69)	21, 3.25 (2.03)	20, 3.74 (1.86)
Timed Up and Go (secs) [a]	23, 27.57 (27.57)	21, 21.24 (11.18)	21, 20.76 (19.64)	19, 16.37 (9.69)	21, 20.76 (19.25)	20, 15.95 (12.00)
Physical Activity: **Diary***	21, 6.67 (19.20)	20, 10.69 (17.39)	21, 17.39 (24.28)	19, 25.60 (34.98)	19, 13.92 (22.25)	20, 35.11 (49.70)
Physical Activity (**Accelerometer**): **Total PA minutes***[b]	21, 145.10 (118.27)	20, 165.56 (139.09)	19, 134.88 (129.77)	18, 178.15 (155.07)	16, 152.08 (118.52)	17, 197.42 (144.31)
Physical Activity (**Accelerometer**): **Light PA minutes*** [b]	21, 92.78 (93.15)	20, 110.18 (115.65)	19, 95.67 (105.50)	18, 121.80 (122.46)	16, 99.33 (99.54)	17, 134.54 (126.36)
Physical Activity (**Accelerometer**): **MVPA PA minutes*** [b]	21, 52.32 (68.94)	20, 55.38 (39.66)	19, 39.21 (39.33)	18, 56.35 (51.26)	16, 52.75 (60.03)	17, 62.88 (41.73)
Physical Activity (**Accelerometer**): **Moderate PA minutes*** [b]	21, 50.53 (66.77)	20, 53.38 (37.04)	19 37.73 (37.40)	18, 53.07 (43.99)	16, 51.11 (58.54)	17, 60.93 (40.84)
Physical Activity (**Accelerometer**): **Vigorous PA minutes*** [b]	21, 1.79 (3.85)	20, 2.00 (3.96)	19, 1.48 (2.39)	18, 3.28 (8.14)	16, 1.64 (2.38)	17, 1.94 (2.33)

Note: ^ post randomisation; *Average minutes of physical activity per day; [a]Precision to 10 ms; [b]Measurement recorded 100 times a second (accelerometer set to a sampling frequency of 100 Hz).

For peer review only - http://bmjopen.bmj.com/site/about/guidelines.xhtml

Table 4b: Number, means and standard deviations as a function of trial arm and measurement time point for candidate secondary outcome measures in the ReTrain pilot trial

	Data collection time point					
	Baseline		6 month^		9 month^	
**Measures:** n, Mean (SD)	**ReTrain (N=23)**	**Control (N=22)**	**ReTrain (N=21)**	**Control (N=20)**	**ReTrain (N=21)**	**Control (N=20)**
Fatigue Assessment Scale	23, 27.17 (7.14)	22, 25.49 (7.44)	21, 24.05 (6.26)	19, 24.05 (8.86)	20, 27.45 (5.95)	20, 25.83 (9.14)
Stroke Self-efficacy Questionnaire	22, 72.41 (22.00)	22, 73.46 (17.87)			20, 73.73 (19.63)	20, 74.40 (16.94)
Outcome expectations for exercise Scale (Exercise Beliefs)	23, 3.66 (0.70)	22, 3.78 (0.52)			19, 4.03 (0.59)	19, 3.73 (0.52)
Short self-efficacy for Exercise Scale (Exercise self-efficacy)	23, 3.26 (0.92)	22, 3.32 (0.89)			19, 3.32 (0.89)	18, 3.22 (1.06)
Stroke Quality of Life Scale(Total)	22, 3.31 (0.68)	22, 3.45 (0.69)			20, 3.38 (0.70)	20, 3.63 (0.82)
EQ-5D-5L	22, 0.51 (0.25)	20, 0.55 (0.24)			19, 0.52 (0.24)	20, 0.62 (0.25)
SF-12: Physical Component	21, 33.12 (7.22)	20, 31.83 (6.69)			19, 33.74 (6.44)	19, 33.25 (6.91)
SF-12: Mental Component	21, 50.10 (7.11)	20, 50.68 (7.98)			19, 50.47 (6.51)	19, 48.05 (8.45)
Modified Caregiver Strain Index (Carer Burden)	8, 11.39 (8.03)	10, 7.40 (7.83)			9, 9.89 (7.22)	6, 9.50 (8.92)

Note: ^ post randomisation; grey cells indicate measurement not taken at this time point

For peer review only - http://bmjopen.bmj.com/site/about/guidelines.xhtml

Table 4a and 4b report mean scores across, respectively, candidate primary and secondary outcome measures at each time point of the pilot trial. The trial was not powered to detect differences in outcome between trial arms or over time and so we do not interpret the patterns of means. However, the results clearly demonstrate that we were able to collect the necessary data and retained acceptable completion rates on all measures across all time points of the study. Attrition was lower than the 20% expected. For each outcome measure (except carer burden as not everyone had a carer) we achieved in excess of the 30 cases (i.e. 15 completed measurements per arm) recommended for pilot studies to estimate outcome variance.

Safety

During assessment periods there was one serious but unrelated event in the intervention group (none in the control group) and slightly fewer overall adverse events in the intervention group (Table 5a).

Table 5a: Adverse Events (AE) and Serious Adverse Events (SAE) reported during 6 and 9-month outcome assessment periods for both ReTrain and Control group

	Event Type	Total Events	Attribution				N People Reporting Event
ReTrain (N=21)			Related	Probably Related	Possible Related	Unrelated	
	**AE**	125[a]	6	5	73	41	19
	**SAE**	1[b]	0	0	0	1	1
Control (N=20)	Event Type	Total Events	Related	Probably Related	Possible Related	Unrelated	N People Reporting Event
	**AE**	150[c]	0	0	0	150	19
	**SAE**	0	0	0	0	0	0

[a]: Muscle soreness (n=26); fatigue (n=58); falls (n=12); trips (n=10); other (n=19; including but not limited to: low mood, itchiness, colds, issues with eyesight, cystitis).

[b]: Ambulance conveyance to A&E due to reaction to antibiotics being taken for chest infection.

[c]: Muscle soreness (n=39); fatigue (n=50); falls (n=19); trips (n=12); other (n=30; including but not limited to: low mood, depression, dizzy spells, sore toes, poor memory, colds, poor sleep, loss of sense of smell, issues with eyesight).

For peer review only

Table 5b: Adverse Events (AE) and Serious Adverse Events (SAE) reported during ReTrain programme

	Event Type	Total Events	Attribution				N People Reporting Event
ReTrain (N=21)			Related	Probably Related	Possible Related	Unrelated	
	**AE**	2[a]	7	0	12	3	11
	**SAE**	6[b]	0	1	1	4	5

[a]: Muscle soreness (n=0); fatigue (n=2); falls (n=10); trips (n=1); other (n=9; including but not limited to: fainting; twisted or swollen ankle, suspected TIA (non-confirmed)).

[b]: Urine retention (n=3); black-out/fainted (n=1); renal & heart failure (n=1); TIA (n=1).

For ReTrain only (Table 5b) there were six serious adverse events during the intervention period: four were unrelated, one possibly related (fainted) and one probably related (TIA) to the intervention. Of the 22 adverse events reported, three of them occurred at the venue (1 x fall; 1 x trip; 1 x ankle strain).

Discussion

The ReTrain pilot trial met all its pre-stated feasibility objectives: the intervention, trial design and research processes were acceptable to participants as well as feasible and safe to deliver; we demonstrated feasibility of recruitment (recruiting above our target of 48), and retention (less than 20% attrition). At the point of randomisation we were slightly under target (45/48). However due to high retention we preserved the number of datasets required (30) to calculate sample size estimates. Furthermore, participants were not unduly burdened by study requirements and there were high completion rates for most outcome measures. We also successfully rehearsed procedures for process and health economic evaluations as well as trial governance processes (trial management and independent trial steering meetings) and maintained our strong Patient and Public Involvement. Participant interviews, outcome measurement results and fidelity assessments highlighted refinements that we have already, or can, put in place for a future definitive RCT of ReTrain. For example, we have some new insights into how to enhance delivery by trainers and engagement by participants (e.g. by placing more focus on individually tailored goal setting; stressing goal and homework reviews; better explanation and promotion of the drop-in sessions). These are all relatively small amendments that are likely to enhance the impact of the training programme. Our trial compares favourably with another feasibility RCT assessing the delivery of the Bridges stroke self-management programme[44] which had relatively low recruitment, questions regarding programme delivery in addition to usual

For peer review only - http://bmjopen.bmj.com/site/about/guidelines.xhtml

rehabilitation, and recommendations for further assessment of intervention fidelity. Some of their findings were similar to ReTrain: participants were broadly positive about their programme; health professionals found it acceptable to use and researchers noted the lack of outcome measure sensitivity for detecting change[44].

Limitations and lessons for planning design of a future trial

When planning this study we selected our candidate primary outcome measures on the basis that they were likely to measure improvements that could be attributed to our intervention; our pilot work was therefore to determine acceptability and feasibility (including their psychometric utility) of these measures. However we were not able to identify a clear candidate primary outcome for a definitive RCT from this pilot work. It is possible that an 'activities of daily living' measure (as typically used in rehabilitation studies) may be more useful in a future definitive trial. Identifying robust outcome measures in rehabilitation trials is a common problem[45] compounded by variability in stroke related disability and participants' comorbidities. This pilot trial was not designed (statistically powered) to test for differences between treatment arms, so no inferential analyses were performed. Any perceived trend (or absence of a trend) should not be interpreted as an indication of an effect (or its absence) and outcomes should not be selected based upon any assumed trend. Acceptability outcomes coupled with a pragmatic and efficient (cost-effective) trial design better inform choice of outcome. From our sample the Timed Up and Go task would be unsuitable due to potentially large sample size requirements (~2000 participants) and the baseline high levels of mobility meant the Rivermead Mobility Index demonstrated a ceiling effect, so could only be used if we altered inclusion criteria. Physical activity was measured robustly via accelerometry and may be the best candidate. We had some software and hardware malfunctions but important lessons have been learned to mitigate these problems in future. Capture of frequency and intensity of activity would allow comparison with stroke guidelines. Although there is a cost implication, accelerometry provides a more objective measurement of daily activity and may also be an adequate proxy of functional mobility, however we will also investigate the benefits of using other PA measures such as questionnaires (instead of our diaries) or using multiple measures such as accelerometry and heart rate monitors whilst being aware of problems with compliance and participant burden[46].

Further limitations relate to the lack of validation of our adherence measure and the local demographics: our sample did not have a wide age range or ethnic diversity. Whilst we did demonstrate delivery in different locations in the South West our plans for a larger definitive trial would include a wider demographic from more centres across the UK.

For a future trial we plan to implement more readable, higher quality written (and pictorial) information and questionnaires although the amount of information provided was appropriate. We will mitigate recruitment loss prior to randomisation by establishing expression of interest and eligibility to take part but delaying taking consent until we are

For peer review only - http://bmjopen.bmj.com/site/about/guidelines.xhtml

confident of sufficient numbers to create a cohort for randomisation; this has resource implications that will need to be built into future funding. We will run ReTrain in community centres or halls as these were more acceptable and much cheaper than gyms; we will provide a more detailed ReTrain induction to ensure trainers understand and communicate all components of the programme. For the QALY comparisons recent policy changes mean the conversion from SF-12 to SF-6D has been phased out, and so less justification for using the SF-12 in a future study. Instead we will consider using the Stroke Impact Scale (SIS) as this is a valid health-related QoL measure. This may also be a better candidate self-report primary outcome measure for a definitive trial as it has shown sensitivity in long-term stroke survivors who have mild to moderate stroke[47]. The SIS assesses multiple facets of physical and emotional issues and so would align with perceived physical and psychological benefits participants attribute to ReTrain. Our sample size estimates for candidate objective primary outcomes (Table 2) indicate we will need a moderately sized trial (n=562, effect size 0.3 or n=413, effect size 0.35) for PA assessed by accelerometry or a smaller trial (n=96) if we use the physical component domain of the SIS (based on 80% power, 5% alpha and assuming 20% attrition[48]). We have established appropriate process evaluation methods to capture multiple facets of intervention fidelity.

Generalisability

This pilot study was not designed to demonstrate generalisability however our participant population represent the sub-set of community-dwelling stroke survivors who have some independent mobility but remain with stroke-related disability that affects their QoL. Our participants also represent the growing proportion of people who have more than one long-term condition. ReTrain techniques target the effects of stroke but can accommodate other conditions which trainers take into account when preparing the participant's individually tailored programme. Some of the key ReTrain (ARNI based) techniques are designed for people with unilateral impairment, such as hemiparesis; however one of our participant's main unilateral impairment was due to diabetes related lower limb amputation, illustrating how ReTrain can accommodate people with multiple co-morbidities.

Conclusion

Our pilot trial has demonstrated that ReTrain is feasible, acceptable, and safe. We met our recruitment and retention targets and demonstrated our ability to run our intervention in different locations. Participants were not unduly burdened by study requirements and most outcome measures had high levels of completion. We successfully tested procedures for process and health economic evaluations. Participant interviews, outcome measurement results and fidelity assessments highlighted some issues needing refinement prior to a future definitive RCT of ReTrain. Many of these have already been addressed and we intend to seek funding for a definitive trial.

For peer review only - http://bmjopen.bmj.com/site/about/guidelines.xhtml

Other information

Protocol Version: 5 Date: 20/04/2016. Published version available here: http://bmjopen.bmj.com/content/6/10/e012375.full

Acknowledgements

We thank our funders, the Stroke Association and the Peninsula Patient Involvement Group with the ReTrain Stroke Service User Group for their help. The National Institute for Health Research (NIHR) Collaboration for Leadership in Applied Health Research and Care South West Peninsula at the Royal Devon and Exeter NHS Foundation Trust also supported this work but views expressed are those of the author(s) and not necessarily those of the NHS, the NIHR or the Department of Health. We also thank our Trial Steering Committee: Ailie Turton (University of the West of England) Siobhan Creanor (Plymouth University), Debbie Neal (Bournemouth University), Justin Smallwood (Patient and Public representative), and Gail Seymour (University of Exeter - Sponsor).

Ethical review by NRES Committee South West – Cornwall & Plymouth (REC ref: 15/SW/04).

Author's Contributions

SGD led the team and drafted this article, RC prepared protocol, ethical submission and amendments, managed the project, contributed to analysis; LP drafted protocol prior to funding application, conducted interviews, contributed to analysis, AF, MJ, RA, MN, SGD & LP provided stroke rehabilitation expertise; RST provided statistical and trial methodological expertise, led analysis; MN provided qualitative expertise and analysed qualitative data; AIS led accelerometry work, supported by RP who provided accelerometry analysis; SGD & LP provided process evaluation expertise, SGD led the process evaluation and supervised LH; LH led video analysis work; AS provided health economic expertise and led economic work supported by PL; SS provided patient and public involvement expertise. All authors commented on the manuscript.

Data sharing statement: Participants did not consent for datasets to be stored or accessed outside of the research team. Therefore no datasets have been made publicly available.

Competing Interests: We declare funding from the Stroke Association.

For peer review only

For peer review only - http://bmjopen.bmj.com/site/about/guidelines.xhtml

References

1. Feigin VL, Barker-Collo S, McNaughton H, et al. Long-term neuropsychological and functional outcomes in stroke survivors: current evidence and perspectives for new research. *Int J Stroke* 2008;3(1):33-40.
2. Townsend N, Wickramasinghe K, Bhatnagar P, et al. Coronary heart disease statistics: 2012 edition. London: British Heart Foundation, 2012.
3. Teasell R, Mehta S, Pereira S, et al. Time to rethink long-term rehabilitation management of stroke patients. *Top Stroke Rehabil* 2012;19(6):457-62.
4. McKevitt C, Fudge N, Redfern J, et al. Self-reported long-term needs after stroke. *Stroke* 2011;42(5):1398-403.
5. Intercollegiate Stroke Working Party. National Clincial Guideline for Stroke, Fifth Edition. 5 ed. London: *Royal College of Physicians* 2016.
6. Rand D, Eng JJ, Tang PF, et al. How active are people with stroke?: use of accelerometers to assess physical activity. *Stroke* 2009;40(1):163-8.
7. Stroke Association. Stroke Statistics. London: Stroke Association, 2013.
8. Best C, van Wijck F, Dennis J, et al. A survey of community exercise programmes for stroke survivors in Scotland. *Health Soc Care Community* 2012;20(4):400-11.
9. NHS Stroke Improvement Programme. Life after stroke: Participating in community exercise and fitness 2012 [Available from: http://www.improvement.nhs.uk/stroke/CommunityStrokeResource/CSRLifeafterstroke/CSRLifeafterstrokeexercisepoststroke/tabid/226/Default.aspx accessed July 2012.
10. Harrington R, Taylor G, Hollinghurst S, et al. A community-based exercise and education scheme for stroke survivors: a randomized controlled trial and economic evaluation. *Clin Rehabil* 2010;24(1):3-15.
11. French B, Thomas LH, Leathley MJ, et al. Repetitive task training for improving functional ability after stroke. *Cochrane Database Syst Rev* 2007(4):Cd006073.
12. Lennon S, McKenna S, Jones F. Self-management programmes for people post stroke: a systematic review. *Clin Rehabil* 2013;27(10):867-78.
13. Brazzelli M, Saunders David H, Greig Carolyn A, et al. Physical fitness training for stroke patients. *Cochrane Database Syst Rev* 2011; (11). http://www.mrw.interscience.wiley.com/cochrane/clsysrev/articles/CD003316/frame.html.
14. Balchin T. The Successful Stroke Survivor: A new guide to functional recovery from stroke. Lingfield, UK: ARNI Trust 2011.
15. Poltawski L. Survey of Group ARNI classes in England, 2011, unpublished report.
16. Craig P, Dieppe P, Macintyre S, et al. Developing and evaluating complex interventions: the new Medical Research Council guidance. *BMJ* 2008;337:a1655.
17. Poltawski L, Abraham C, Forster A, et al. Synthesising practice guidelines for the development of community-based exercise programmes after stroke. *Implement Sci* 2013;8:115.
18. Kilbride C, Norris M, Theis N, et al. Action for Rehabilitation from Neurological Injury (ARNI): A pragmatic study of functional training for stroke survivors. *Open J Ther Rehabil* 2013;1:40-51.
19. Poltawski L, Briggs J, Forster A, et al. Informing the design of a randomised controlled trial of an exercise-based programme for long term stroke survivors: lessons from a before-and-after case series study. *BMC Res Notes* 2013;6:324.

For peer review only - http://bmjopen.bmj.com/site/about/guidelines.xhtml

20. Norris M, Kilbride C, Mohagheghi A, et al. A Qualitative Exploration of Participation in an Exercise Instructor Led Functional Training Programme for Community Dwelling Stroke Survivors. *Int J Ther Rehabil* 2013;20(12):597-605.
21. Eldridge SM, Chan CL, Campbell MJ, et al. CONSORT 2010 statement: extension to randomised pilot and feasibility trials. *BMJ* 2016;355.
22. Dean SG, Poltawski L, Forster A, et al. Community-based Rehabilitation Training after stroke: protocol of a pilot randomised controlled trial (ReTrain). *BMJ Open* 2016;6(10).
23. Stroke Association. Exercise and stroke London2013 [Available from: https://www.stroke.org.uk/sites/default/files/exercise_and_stroke.pdf accessed 24 05 2017 2017].
24. ACSM. American College of Sports Medicine Guidelines for Exercise Testing and Prescription. Philadelphia: American College of Sports Medicine, 2005.
25. Carnes D, Mullinger B, Underwood M. Defining adverse events in manual therapies: A modified Delphi consensus study. *Man Ther* 2010;15(1);2-6.
26. Green J, Forster A, Young J. A test-retest reliability study of the Barthel Index, the Rivermead Mobility Index, the Nottingham Extended Activities of Daily Living Scale and the Frenchay Activities Index in stroke patients. *Disabil Rehabil* 2001;23(15):670-6.
27. Hsieh CL, Hsueh IP, Mao HF. Validity and responsiveness of the rivermead mobility index in stroke patients. *Scand J Rehabil Med* 2000;32(3):140-2.
28. Ng SS, Hui-Chan CW. The timed up & go test: its reliability and association with lower-limb impairments and locomotor capacities in people with chronic stroke. *Arch Phys Med Rehabil* 2005;86(8):1641-7.
29. Stratford P, Gill C, Westaway M, et al. Assessing Disability and Change on Individual Patients: A Report of a Patient Specific Measure. *Physiother Can* 1995;47(4):258-63.
30. Jones F, Partridge C, Reid F. The Stroke Self-Efficacy Questionnaire: measuring individual confidence in functional performance after stroke. *J Clin Nurs* 2008;17(7b):244-52.
31. Michielsen HJ, De Vries J, Van Heck GL. Psychometric qualities of a brief self-rated fatigue measure: The Fatigue Assessment Scale. *J Psychosom Res* 2003;54(4):345-52.
32. Mead G, Lynch J, Greig C, et al. Evaluation of fatigue scales in stroke patients. *Stroke* 2007;38(7):2090-5.
33. Shaughnessy M, Resnick BM, Macko RF. Reliability and validity testing of the short self-efficacy and outcome expectation for exercise scales in stroke survivors. *J Stroke Cerebrovasc Dis* 2004;13(5):214-9.
34. Bohannon RW, Maljanian R, Lee N, et al. Measurement properties of the short form (SF)-12 applied to patients with stroke. *Int J Rehabil Res* 2004;27(2):151-4.
35. Herdman M, Gudex C, Lloyd A, et al. Development and preliminary testing of the new five-level version of EQ-5D (EQ-5D-5L). *Qual Life Res* 2011;20(10):1727-36.
36. Williams LS, Weinberger M, Harris LE, et al. Development of a stroke-specific quality of life scale. *Stroke* 1999;30(7):1362-9.
37. Thornton M, Travis SS. Analysis of the reliability of the modified caregiver strain index. *J Gerontol B, Psychol Sci Soc Sci* 2003;58(2):S127-32.
38. Craig LE, Wu O, Bernhardt J, et al. Approaches to economic evaluations of stroke rehabilitation. *Int J Stroke* 2014;9(1):88-100.
39. Browne RH. On the use of a pilot sample for sample size determination. *Stat Med* 1995;14(17):1933-4.

For peer review only

For peer review only - http://bmjopen.bmj.com/site/about/guidelines.xhtml

40. Bruno A, Akinwuntan AE, Lin C, et al. Simplified Modified Rankin Scale Questionnaire: Reproducibility Over the Telephone and Validation With Quality of Life. *Stroke* 2011; 42(8)2276-9.

41. Hislop J, Adewuyi TE, Vale LD, et al. Methods for specifying the target difference in a randomised controlled trial: the Difference ELicitation in TriAls (DELTA) systematic review. *PLoS Med* 2014;11(5):e1001645.

42. Gautschi OP, Stienen MN, Corniola et al. Assessment of the Minimum Clinically Important Difference in the Timed Up and Go Test After Surgery for Lumbar Degenerative Disc Disease. *Neurosurgery* 2017; 80 (3):380-385.

43. Horn KK, Jennings S, Richardson G, et al. The patient-specific functional scale: psychometrics, clinimetrics, and application as a clinical outcome measure. *J Orthop Sports Phys Ther* 2012; 42 (1):30-42.

44. McKenna S, Jones F, Glenfield P, et al. Bridges self-management program for people with stroke in the community: A feasibility randomized controlled trial. *Int J Sroke* 2015;10(5):697-704.

45. Wade DT. Outcome measures for clinical rehabilitation trials: impairment, function, quality of life, or value? *Am J Phys Med Rehabil* 2003;82(10 Suppl):S26-31.

48. Mansfield A, Knorr S, Poon V, et al. Promoting Optimal Physical Exercsie for Life: An Exercise and Self-Management Program to Encourage Participation in Physical Activity after Discharge from Stroke Rehabilitation – A Feasibility Study. *Stroke Research and Treatment* 2016; [Available from http://dx.doi.org/10.1155/2016/9476541].

47. Rehabilitation Institute of Chicago. Rehabilitation Measures Database - Stroke Impact Scale [Available from: http://www.rehabmeasures.org/Lists/RehabMeasures/DispForm.aspx?ID=934 accessed 21 06 2017].

48. Saywell N, Vandal AC, Brown P, et al. Telerehabilitation to improve outcomes for people with stroke: study protocol for a randomised controlled trial. *Trials* 2012;13(1):233.

For peer review only

For peer review only - http://bmjopen.bmj.com/site/about/guidelines.xhtml

Figure 1 – Recruitment and randomisation by cohort

For peer review only

For peer review only - http://bmjopen.bmj.com/site/about/guidelines.xhtml

Figure 2 – Participant flow through the trial

For peer review only

For peer review only - http://bmjopen.bmj.com/site/about/guidelines.xhtml

Figure 1 – Recruitment and randomisation by cohort

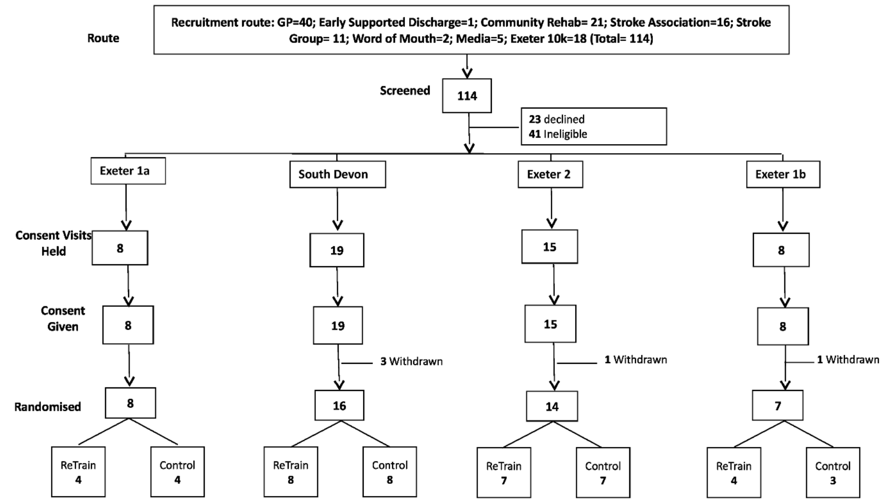

Figure 1 – Recruitment and randomisation by cohort

297x209mm (300 x 300 DPI)

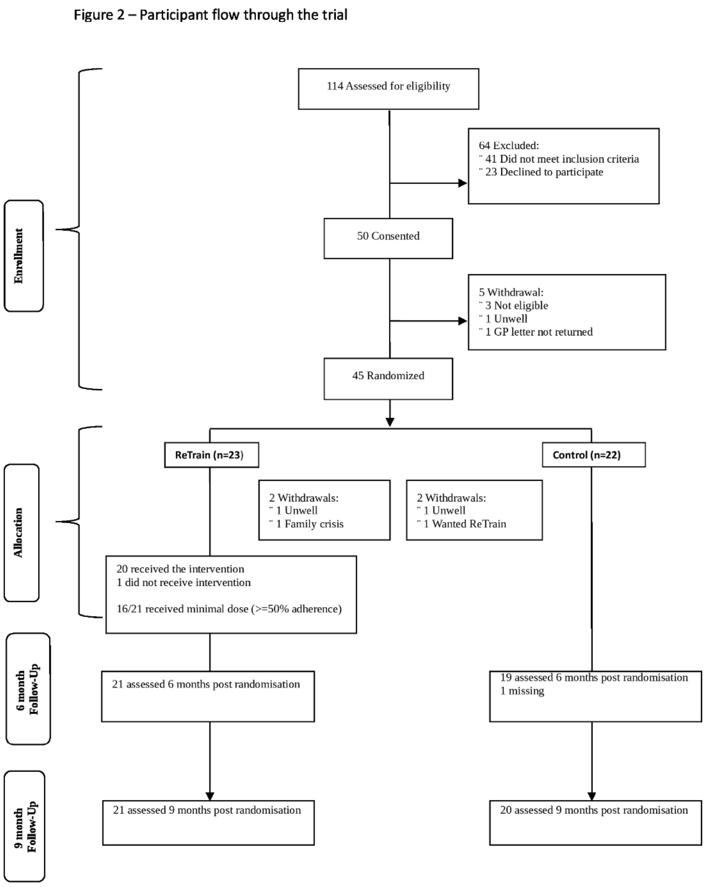

Figure 2 – Participant flow through the trial

209x297mm (300 x 300 DPI)

Table 2 | CONSORT checklist of information to include when reporting a pilot trial

Section/topic and item No	Standard checklist item	Extension for pilot trials	Page No where item is reported
**Title and abstract**			
1a	Identification as a randomised trial in the title	Identification as a pilot or feasibility randomised trial in the title	1
1b	Structured summary of trial design, methods, results, and conclusions (for specific guidance see CONSORT for abstracts)	Structured summary of pilot trial design, methods, results, and conclusions (for specific guidance see CONSORT abstract extension for pilot trials)	2
**Introduction**			
Background and objectives:			
2a	Scientific background and explanation of rationale	Scientific background and explanation of rationale for future definitive trial, and reasons for randomised pilot trial	4
2b	Specific objectives or hypotheses	Specific objectives or research questions for pilot trial	4-5
**Methods**			
Trial design:			
3a	Description of trial design (such as parallel, factorial) including allocation ratio	Description of pilot trial design (such as parallel, factorial) including allocation ratio	5
3b	Important changes to methods after trial commencement (such as eligibility criteria), with reasons	Important changes to methods after pilot trial commencement (such as eligibility criteria), with reasons	N/A
Participants:			
4a	Eligibility criteria for participants		5
4b	Settings and locations where the data were collected		5-6
4c		How participants were identified and consented	5
Interventions:			
5	The interventions for each group with sufficient details to allow replication, including how and when they were actually administered		5-6
Outcomes:			
6a	Completely defined prespecified primary and secondary outcome measures, including how and when they were assessed	Completely defined prespecified assessments or measurements to address each pilot trial objective specified in 2b, including how and when they were assessed	6
6b	Any changes to trial outcomes after the trial commenced, with reasons	Any changes to pilot trial assessments or measurements after the pilot trial commenced, with reasons	N/A
6c		If applicable, prespecified criteria used to judge whether, or how, to proceed with future definitive trial	N/A
Sample size:			
7a	How sample size was determined	Rationale for numbers in the pilot trial	6-7
7b	When applicable, explanation of any interim analyses and stopping guidelines		N/A
Randomisation:			
Sequence generation:			
8a	Method used to generate the random allocation sequence		7
8b	Type of randomisation; details of any restriction (such as blocking and block size)	Type of randomisation(s); details of any restriction (such as blocking and block size)	7
Allocation concealment mechanism:			
9	Mechanism used to implement the random allocation sequence (such as sequentially numbered containers), describing any steps taken to conceal the sequence until interventions were assigned		7
Implementation:			
10	Who generated the random allocation sequence, enrolled participants, and assigned participants to interventions		7
Blinding:			
11a	If done, who was blinded after assignment to interventions (eg, participants, care providers, those assessing outcomes) and how		7
11b	If relevant, description of the similarity of interventions		N/A
Analytical methods:			
12a	Statistical methods used to compare groups for primary and secondary outcomes	Methods used to address each pilot trial objective whether qualitative or quantitative	7
12b	Methods for additional analyses, such as subgroup analyses and adjusted analyses	Not applicable	N/A

For peer review only - http://bmjopen.bmj.com/site/about/guidelines.xhtml

RESEARCH METHODS AND REPORTING

Table 2 | CONSORT checklist of information to include when reporting a pilot trial

Section/topic and item No	Standard checklist item	Extension for pilot trials	Page No where item is reported
**Results**			
Participant flow (a diagram is strongly recommended):			
13a	For each group, the numbers of participants who were randomly assigned, received intended treatment, and were analysed for the primary outcome	For each group, the numbers of participants who were approached and/or assessed for eligibility, randomly assigned, received intended treatment, and were assessed for each objective	Figure 1a & 1b & P.8
13b	For each group, losses and exclusions after randomisation, together with reasons		Figure 1a & 1b & p.10
Recruitment:			
14a	Dates defining the periods of recruitment and follow-up		8
14b	Why the trial ended or was stopped	Why the pilot trial ended or was stopped	N/A
Baseline data:			
15	A table showing baseline demographic and clinical characteristics for each group		8 – 10
Numbers analysed:			
16	For each group, number of participants (denominator) included in each analysis and whether the analysis was by original assigned groups	For each objective, number of participants (denominator) included in each analysis. If relevant, these numbers should be by randomised group	Figure 1b
Outcomes and estimation:			
17a	For each primary and secondary outcome, results for each group, and the estimated effect size and its precision (such as 95% confidence interval)	For each objective, results including expressions of uncertainty (such as 95% confidence interval) for any estimates. If relevant, these results should be by randomised group	N/A
17b	For binary outcomes, presentation of both absolute and relative effect sizes is recommended	Not applicable	N/A
Ancillary analyses:			
18	Results of any other analyses performed, including subgroup analyses and adjusted analyses, distinguishing prespecified from exploratory	Results of any other analyses performed that could be used to inform the future definitive trial	10 – 15
Harms:			
19	All important harms or unintended effects in each group (for specific guidance see CONSORT for harms)		16
19a		If relevant, other important unintended consequences	N/A
**Discussion**			
Limitations:			
20	Trial limitations, addressing sources of potential bias, imprecision, and, if relevant, multiplicity of analyses	Pilot trial limitations, addressing sources of potential bias and remaining uncertainty about feasibility	17 – 18
Generalisability:			
21	Generalisability (external validity, applicability) of the trial findings	Generalisability (applicability) of pilot trial methods and findings to future definitive trial and other studies	18
Interpretation:			
22	Interpretation consistent with results, balancing benefits and harms, and considering other relevant evidence	Interpretation consistent with pilot trial objectives and findings, balancing potential benefits and harms, and considering other relevant evidence	18 – 19
22a		Implications for progression from pilot to future definitive trial, including any proposed amendments	19
**Other information**			
Registration:			
23	Registration number and name of trial registry	Registration number for pilot trial and name of trial registry	2, 19
Protocol:			
24	Where the full trial protocol can be accessed, if available	Where the pilot trial protocol can be accessed, if available	19
Funding:			
25	Sources of funding and other support (such as supply of drugs), role of funders		2, 19
26		Ethical approval or approval by research review committee, confirmed with reference number	19

For peer review only - http://bmjopen.bmj.com/site/about/guidelines.xhtml

BMJ Open

Community-based Rehabilitation Training after stroke: Results of a pilot randomised controlled trial (ReTrain) investigating acceptability and feasibility

Journal:	*BMJ Open*
Manuscript ID	bmjopen-2017-018409.R2
Article Type:	Research
Date Submitted by the Author:	20-Sep-2017
Complete List of Authors:	Dean, Sarah; PenCLAHRC University of Exeter Medical School,<br>Poltawski, Leon; University of Exeter Medical School<br>Forster, Anne; University of Leeds, Academic Unit of Elderly Care and Rehabilitat<br>Taylor, Rod; University of Exeter, Peninsula Medical School<br>Spencer, Anne<br>James, Martin; South West NHS Trust<br>Allison, Rhoda; Torbay and Southern Devon Health and Care NHS Trust<br>Stevens, Shirley; University of Exeter Medical School<br>Norris, Meriel; Brunel University London<br>Shepherd, Anthony; University of Portsmouth, Sport and Exercise Science<br>Landa, P; University of Exeter, Medical School<br>Pulsford, Richard; University of Exeter, Sports and Health Science<br>Hollands, Laura; University of Exeter Medical School<br>Calitri, Raff; University of Exeter Medical School
**Primary Subject Heading**:	Rehabilitation medicine
Secondary Subject Heading:	Cardiovascular medicine, Health services research
Keywords:	STROKE MEDICINE, REHABILITATION MEDICINE, Clinical trials < THERAPEUTICS

SCHOLARONE™
Manuscripts

Title Community-based Rehabilitation Training after stroke: Results of a pilot randomised controlled trial (ReTrain) investigating acceptability and feasibility

Sarah G Dean[1], Leon Poltawski[1], Anne Forster[2], Rod S Taylor[1], Anne Spencer[1], Martin James[1,3], Rhoda Allison[4], Shirley Stevens[1], Meriel Norris[5], Anthony I Shepherd[6], Paolo Landa[1], Richard M Pulsford[7], Laura Hollands[1], Raff Calitri[1].

[1]University of Exeter Medical School, Exeter, EX1 2LU, UK.

[2]Academic Unit of Elderly Care and Rehabilitation, University of Leeds, Bradford, BD9 6RJ, UK.

[3]Royal Devon & Exeter Hospital, Exeter, EX2 5DW, UK.

[4]Torbay and South Devon NHS Foundation Trust, Torquay, TQ2 7TD, UK.

[5]Department of Clinical Sciences, Brunel University London, Middlesex UB8 3PH, UK.

[6] Department of Sport and Exercise Science, University of Portsmouth, Portsmouth, PO1 2ER.

[7]Sports & Health Sciences, University of Exeter, Exeter, EX1 2LU, UK.

Corresponding author

Associate Professor Sarah Dean,

Psychology Applied to Rehabilitation and Health,

University of Exeter Medical School,

College House

St Luke's Campus,

Exeter,

EX1 2LU,

Email: s.dean@exeter.ac.uk

01392 722984

Word count 4493 exc tables

For peer review only

For peer review only - http://bmjopen.bmj.com/site/about/guidelines.xhtml

Key words: Stroke, Rehabilitation Medicine, Clinical Trials.

Abstract

Objectives: To assess acceptability and feasibility of trial processes and the ReTrain intervention including an assessment of intervention fidelity. **Design:** A two-group, assessor-blinded, randomised controlled trial with parallel mixed methods process and economic evaluations**. Setting:** Community settings across two sites in Devon. **Participants**: Eligible participants were: 18 years old or over, with a diagnosis of stroke and with self-reported mobility issues, no contraindications to physical activity, discharged from National Health Service (NHS) or any other formal rehabilitation programme at least 1 month prior, willing to be randomised to either control or ReTrain and attend the training venue, possessing cognitive capacity and communication ability sufficient to participate. Participants were individually randomised (1:1) via a computer generated randomisation sequence minimised for time since stroke and level of functional disability. Only outcome assessors independent of the research team were blinded to group allocation. **Interventions:** ReTrain comprised (1) an introductory one-to-one session; (2) ten, twice weekly group classes with up to two trainers and eight clients; (3) a closing one-to-one session, followed by three drop-in sessions over the subsequent three months. Participants received a bespoke home-based training programme. All participants received treatment as usual. The control group received an exercise after stroke advice booklet. **Outcome measures:** Candidate primary outcomes included functional mobility and physical activity. **Results:** Forty-five participants were randomised (ReTrain=23; Control=22); data were available from 40 participants at six months follow-up (ReTrain=21; Control=19) and 41 at nine months follow-up (ReTrain=21; Control=20). We demonstrated ability to recruit and retain participants. Participants were not burdened by the requirements of the study. We were able to calculate sample estimates for candidate primary outcomes and test procedures for process and health economic evaluations. **Conclusions:** All objectives were fulfilled and indicated that a definitive trial of ReTrain is feasible and acceptable.

Registration: ClinicalTrials.gov: trial number NCT02429180.

Funding: The Stroke Association TSA 2014-13

For peer review only

For peer review only - http://bmjopen.bmj.com/site/about/guidelines.xhtml

Strengths and limitations of this study

- A community-based exercise intervention after stroke developed with service users
- A pilot randomised trial developed following MRC guidelines on complex interventions
- Mixed-method approach for answering feasibility and acceptability objectives
- Objective physical activity capture via accelerometry but subjective self-report outcomes for functional mobility and psycho-social measurements

For peer review only

For peer review only - http://bmjopen.bmj.com/site/about/guidelines.xhtml

Introduction

Five years after initial stroke, one in three individuals have residual physical impairment[1], equating to over 300,000 individuals in the United Kingdom (UK) living with disability from stroke[2]. Provision of stroke rehabilitation is typically front loaded, with resources focussed on in-patient care and early supported discharge. Support tapers off after a few months[3] with many individuals reporting unmet long-term needs[4].

The National Clinical Guideline for Stroke advise for secondary prevention that stroke survivors engage in 150 minutes of physical activity a week, in bouts of 10 minutes or more, starting light and developing across time to moderate levels of intensity[5]. However, many stroke survivors do not meet these recommendations[6 7] due to combinations of personal (e.g., physical or psychological impairments) and environmental factors (e.g., lack of programmes and facilities). To address this problem, community-based programmes are promoted[8-10]. These tend to focus on cardiovascular fitness with less emphasis on functional improvements or on promoting on-going exercise self-management. National stroke guidelines[5] identify the importance of interventions for functional improvement[11] and self-management[12] but evidence is lacking regarding these types of intervention[13].

Action for Rehabilitation following Neurological Injury (ARNI) is an approach aimed at improving function and facilitating self-management[14] and has a detailed self-help book. The ARNI approach embodies a set of principles (e.g. instilling a commitment to regular exercise) and techniques tailored to individual need. The ARNI Institute trains registered exercise professionals to deliver key ARNI techniques. Clinical Commissioning Groups (CCGs), charitable, and local authorities have started to provide community-based ARNI training for stroke survivors, which has been positively received by participants, carers and practitioners[15], however there is currently no randomised controlled trial (RCT) evidence for evaluating its impact on stroke outcomes or its cost-effectiveness.

Background and objectives

Using the Medical Research Council's framework for the development and evaluation of complex interventions[16] and considerable Patient and Public Involvement we have designed a testable programme called Rehabilitation Training (ReTrain)[17-20]. ReTrain is a community-based, manualised group programme combining ARNI principles and key techniques with best practice guidelines for stroke[9 17]. The overall aim of our pilot RCT was to inform the design and delivery of a definitive RCT. Our objectives were to: 1) assess feasibility and acceptability of recruitment (target n=48), randomisation, allocation concealment and outcome assessment blinding; 2) determine retention rates (target of less than 20% attrition); 3) check ReTrain's acceptability and feasibility for participants, and refine the Trainer Manual; 4) test candidate outcome measures, assess their burden, levels of completion, and estimate outcome variance (to inform definitive trial sample size); 5)

For peer review only - http://bmjopen.bmj.com/site/about/guidelines.xhtml

perform process evaluation including intervention fidelity assessment; 6) calculate ReTrain costs and assess feasibility of collecting health and social service resource use.

Methods

A brief methods overview is provided in accordance with guidance for reporting pilot trials[21]; further details are available in the published protocol[22]. Ethics review was conducted by National Research Ethics Service Committee South West Cornwall & Plymouth (REC Ref: 15/SW/04).

Trial design

ReTrain was a two-group, assessor-blinded, randomised controlled external pilot trial with parallel mixed methods process and economic evaluations. Eligible participants were individually randomised 1:1 to intervention (ReTrain) or control (exercise advice booklet[23]).

Participants

Inclusion criteria were: i) diagnosis of stroke; ii) any time since stroke but at least 1 month since discharge from NHS physical rehabilitation services; iii) able to walk independently indoors with or without mobility aids, but with self-reported difficulty with stairs, slopes or uneven surfaces; iv) willingness to be randomised and attend the training venue; v) cognitive capacity and communication ability sufficient to participate.

Exclusion criteria were: less than 18 years old, currently (or within one month of) receiving ARNI training or have contraindications to moderate to vigorous physical activity (adapted from American College of Sports Medicine guidelines[24]). Participants were recruited from two CCGs. Participants were identified by: (1) clinicians in NHS primary care, hospital and community stroke services; (2) contacts in the local Clinical Research Network and Clinical Research Facility; (3) promotion via local stroke support networks (e.g. Stroke Association); (4) word of mouth, study flyers and adverts.

Intervention

ReTrain aims to: (1) enhance function through task-related practice, teaching compensatory techniques, and providing targeted strength training (cardiovascular fitness gains also occur through these activities); (2) develop self-management skills for on-going rehabilitation; (3) deliver personalised training using negotiated goals and (4) instil a commitment to regular exercise for health improvement and longer-term maintenance. ReTrain facilitates safe and efficient practice of walking in varied terrains, kerbs, cambers and in crowds, turning and moving quickly, climbing steps and stairs without rails, getting to and from the floor without furniture or other aids, and moving without mobility aids or while carrying loads. Training is based on a manual and led by personal trainers on the UK Register of Exercise Professionals (level 3 or above) who are ARNI-trained and accredited and have had additional training in the delivery of ReTrain. There was a maximum ratio of one trainer to four stroke survivors.

For peer review only - http://bmjopen.bmj.com/site/about/guidelines.xhtml

For peer review only

ReTrain was delivered in a community setting (one gym, two church halls, one community centre) with twice weekly two hour sessions over three months, comprising: an introductory one-to-one session (home visit); ten, twice-weekly group classes with up to two trainers and eight clients (training venue); a closing one-to-one session (home visit); followed by three (one per month) drop-in sessions. Participants completed bespoke home-based training (homework) throughout.

Control

All participants received treatment as usual. This ranged from zero treatment to engagement with any health service(s). We requested that all trial participants did not participate in additional physical rehabilitation (either NHS or private) but we could not prevent them from doing so. We did not monitor control group participation in any treatments during the trial but did record health service use at the end of the trial for all participants. The control group also received an advice booklet about exercise after stroke[23].

Outcomes

Feasibility, acceptability and process outcomes: numbers and details of those approached; recruitment and retention figures. ***Acceptability of randomisation, outcome measurement burden, and the intervention:*** completion of questionnaires and objective assessments; interviews with ten intervention and ten control group members, and the trainers. ***Safety****:* Adverse events[25] identified via trainer and ReTrain participants (during the programme) and participant reports (all participants during 6 and 9-month assessments). ***Intervention fidelity:*** attendance registers, accelerometry, exercise 'homework' diaries, trainer completed session checklists and video analysis of (early, middle and late programme) training sessions.

We tested a range of candidate primary and secondary outcome measures. ***Primary Outcomes***: Rivermead Mobility Index[26 27]; Timed Up and Go Test[28]; modified Patient-Specific Functional Scale[29]; 7-day objective physical activity levels using wrist-worn accelerometry (GENEActiv, Activinsights, Kimbolton, Cambridge UK) and a physical activity diary. ***Secondary Outcomes***: Stroke Self-efficacy Questionnaire[30]; Fatigue Assessment Scale[31 32]; exercise beliefs and exercise self-efficacy questionnaires[33]; SF12[34]; EQ-5D-5L[35]; Stroke Quality of Life (QoL) questionnaires[36]; Carer Burden Index[37]; and Health and Social Service use through a Service Receipt Inventory[38].

Physical outcome baseline assessments (completed by research team) and follow-up assessments (at 6 and 9-months, completed by blinded assessor) were conducted in the participant's home. Researchers visited participants to fit the accelerometer, drop off questionnaires and diary one week prior to blind assessor visits. Assessors administered primary outcome physical measures and collected accelerometers, questionnaires and diaries.

For peer review only - http://bmjopen.bmj.com/site/about/guidelines.xhtml

Sample size

We required 48 participants (24 per group) as (a) 30 complete data sets are recommended for pilot studies to estimate outcome variance[39] and (b) we wanted to investigate variations in context by running the intervention three times (*i.e.* 3 x 8 patients). This number also allowed estimation of a predicted attrition rate of 20% with a precision of ± 5% with 95% certainty.

Randomisation and blinding

The random sequence was computer generated with minimisation for time since stroke (≤ 3 months versus > 3 months) and level of functional disability (modified Rankin Scale (mRS)[40] score ≤ 2 versus > 2). Allocation concealment was ensured by using a password protected validated web-based remote randomisation service supported by the Peninsula Clinical Trials Unit (PenCTU). The Trial Manager requested randomisation only after a cohort of participants had been consented.

Participants, trainers providing the intervention, and researchers conducting the process and economic evaluations could not be blinded to allocation. However, outcomes were assessed by independent researchers (not based at research centre) who were blinded to group allocation. Participants were reminded not to reveal their allocation to assessors but any un-blinding was recorded; after assessments assessors were asked to guess participant allocation.

Data Analysis

Analysis was primarily descriptive with participant flow summarised and estimates of screening, recruitment and attrition reported. Means and standard deviations for all outcomes are reported at baseline, 6 and 9-months follow-up for each group.

Intervention fidelity was assessed using mixed methods: qualitative video analysis comparing the Trainer Manual standard versus observed technique (two researchers independently assessed videos) combined with interview data and summary scores from trainer completed session checklists. Qualitative data were manually analysed descriptively and with content analysis for trial processes; additional thematic analysis was used for interview data. One person (MN) led the qualitative analysis but this was then discussed (MN & SD), checked (RC) and agreed (MN, SD, LP, RC).

We used a micro-costing approach to calculate costs associated with ReTrain: staff time (trainers, administrator, facilitators), venue hire, training equipment (annualised over time), course materials, consumables, travel costs (participants, trainers and facilitators). The costs of the intervention were estimated as a cost per programme and a cost per participant. The estimated costs of the intervention per participant were based on the number of participants enrolled on the programme. The base case scenario assumed the average

For peer review only - http://bmjopen.bmj.com/site/about/guidelines.xhtml

number of participants per programme across all cohorts. Sensitivity analyses were conducted using the minimum and maximum number of participants enrolled for the programme and the quantity of programme materials that were wasted. We analysed the relative benefits of calculating health related QoL using SF-6D (developed from the SF-12) over the QALY calculated (using EQ-5D 5L) from the baseline measures.

Sample size estimates for a definitive trial were calculated for candidate primary outcomes using the standard deviation observed in this pilot population and published minimal clinical important difference (MCID) at 90% power and 5% alpha, and assuming 20% attrition. Where no published MCID could be sourced, we assumed a small to moderate effect size of 0.4 of a standard deviation[41]. The trial statistician undertook calculations using the 'samspi' command in STATA v14.2

Results

Recruitment took place from June 2015 to January 2016. The intervention ran in four cohorts, participant flows are shown for each (Figure 1) and for the trial overall (Figure 2). Initial recruitment was slow so to prevent late running of the trial we split the first cohort. Six-month follow-up outcome assessments took place January to July 2016 and 9-month follow-up April to October 2016.

Objective 1: *Assess the feasibility and acceptability of recruitment, randomisation, allocation concealment and processes for outcome assessment and blinding*

We screened 115 individuals to recruit 50 participants (Figure 1) in 8 months (2 months ahead of schedule). Of these, 45 (90%) were randomised (Figure 1 and 2). Five individuals withdrew prior to randomisation due to ill health or the time lag between agreeing to take part and a cohort being ready to randomise. Table 1 shows baseline characteristics of those randomised, indicating a balance of characteristics across trial arms.

Table 1 Baseline participant demographics

	ReTrain (N= 23)	Control (N=22)
**Gender, n**		
Male (%)	16 (70%)	14 (67%)
**Age (years): mean (SD)**	70 (12)	71 (10)
**Age Category (N=45): n (%)**		
<45	1 (4%)	0 (%)
46-50	0 (0%)	1 (5%)
51-60	3 (13%)	2 (9%)
61-70	10 (43%)	6 (27%)
71-80	5 (22 %)	8 (36%)
81-90	2 (9%)	5 (23%)
90+	2 (9%)	0 (0%)
**Time Since Stroke (no. months):**		

For peer review only - http://bmjopen.bmj.com/site/about/guidelines.xhtml

< 12	3 (13%)	3 (14%)
12-24	4 (17%)	4 (18%)
25-48	5 (22%)	5 (23%)
49-72	2 (9%)	5 (23%)
73-96	4 (17%)	2 (9%)
97+	5 (22%)	3 (14%)
**Time Since Stroke Minimisation Categories (months):n, (%)**		
<=3 months	1 (4%)	0 (0%)
>3 months	22 (96%)	22 (100%)
**Type of Stroke, n (%)**		
Haemorrhagic	3 (13%)	1 (5%)
Ischaemic	15 (65%)	15 (68%)
Both	0 (0%)	1 (5%)
Missing	5 (22%)	5 (23%)
**Stroke Rehabilitation (weeks):**		
n,<br>Average no. weeks (SD)	21,<br>8 (9)	21,<br>14 (19)
Median no. weeks	6	12
Range	0-32	0-88
Unknown length rehab: n	2	1
**Functional Disability (Simplified Modified Rankin Scale score- sMRS): n (%)**		
0	1 (4%)	0 (0%)
1	2 (9%)	1 (5%)
2	4 (17%)	9 (41%)
3	16 (70%)	12(55%)
**sMRS minimisation categories: n(%)**		
<=2	7 (30%)	10 (45%)
>2	16 (70%)	12 (55%)
**Co-morbidities^, n (%)**		
Hypertension	18 (78%)	18 (82%)
Type 2 Diabetes Mellitus	4 (17%)	4 (18%)
Depression	8 (35%)	5 (23%)
Chronic Kidney Disease	2 (9%)	1 (4%)
Asthma / COPD	4 (17%)	3 (14%)
Other	5 (22%)	3 (14%)
**Medications^, n (%)**		
Diuretics	3 (13%)	1 (5%)
Anticoagulants	8 (35%)	10 (45%)
Antiplatelet	15 (65%)	12 (55%)
Antihypertensives<br>Calcium Channel Blockers<br>ACE inhibitors<br>Other	 6 (26%)<br>13 (57%)<br>9 (39%)	 14 (64%)<br>8 (36%)<br>7 (32%)
Statins	18 (78%)	19 (86%)
Anti-depressants	8 (35%)	5 (23%)
Diabetes medication	4 (17%)	4 (18%)

For peer review only

For peer review only - http://bmjopen.bmj.com/site/about/guidelines.xhtml

Chronic pain medication	12 (52%)	8 (36%)
Other	5 (22%)	3 (14%)
**Employment Status, n (%)**		
Employed (and working)	2 (9%)	1 (5%)
Retired	18 (78%)	15 (68%)
Semi-retired	1 (4%)	0 (0%)
Unemployed	2 (9%)	5 (27%)
**Pre-stroke Exercise History, n**		
Exerciser (%)	10 (43%)	8 (36%)
**Mini Mental State Exam:**		
n, Mean (SD)	22*, 27.5 (2.54)	22, 27.9 (3.01)
Median	28	29
Range	19– 30**	19-30**

^ Participants may have more than one co-morbidity / medication.
*1 participant with severe aphasia had difficulties completing the MMSE. The participant could understand and follow instructions and was considered cognitively able to participate in the trial.
**Higher scores indicate better cognitive function. Participants range from no to moderate degree of cognitive impairment.

Blinding of outcome assessors was considered successful as only 2/41 (5%) participants revealed their allocations after completion of outcome measures, both were intervention participants. Different assessors were used for subsequent assessments therefore risk of bias was minimised.

Objective 2: *Acquire retention rates and outcome variance*

Forty out of 45 (88%, 95% CI: 76% to 96%) completed 6-month and 9-month follow-ups. Despite fewer people being randomised than expected, high retention preserved the number of datasets needed to perform our sample size estimates (Table 2).

Table 2 Sample estimates for potential candidate primary outcomes from ReTrain pilot RCT

Primary Outcome Measure	Sample Size Estimates[a]	Minimal Clinically Important Difference (MCID)	Observed SD range	Effect size (MCID/SD)
Rivermead Mobility Index	36 – 44	3.0[b]	2.33 – 2.66	1.13 – 1.29
Timed Up and Go	1,438 – 2,673	1.2 – 3.4[c]	15.69 – 21.39	0.06 – 0.22
Modified Patient Specific Functional Scale	16 – 200	1.0 – 3.0[c]	1.58 – 1.94	0.52 – 1.7

For peer review only - http://bmjopen.bmj.com/site/about/guidelines.xhtml

Physical Activity (Accelerometer)	350 - 1458	Not available	Not applicable	0.2 – 0.45[d]

[a]Figures represent overall (two group) sample size estimates required for a definitive trial. Sample sizes estimated for 1:1 allocation at 90% power and 5% alpha and assuming 20% attrition. Calculations are conservative showing range from best case scenario (largest MCID and smallest SD) to worst case scenario (smallest MCID and largest SD) of SDs observed in this trial and published MCIDs where available.

[b]MCID available from stroke research for the Rivermead Mobility Index (http://www.strokengine.ca/psycho/rmi_psycho/).

[c]MCIDs identified from other disease groups used as proxies as no published stroke MCIDs[42,43].

[d]There are no MCID data available for PA (accelerometry) in stroke (or any other cardio vascular disease) we therefore applied sample size calculations undertaken for a relevant ongoing HTA NIHR trial which estimated n= 562 (effect size 0.3) or n= 413 (effect size 0.35) (http://www.isrctn.com/ISRCTN15644451).

Objective 3: *Check ReTrain's acceptability and feasibility for participants, and refine the Trainer manual*

Eleven themes from 20 qualitative interviews summarise participants' views, Table 3 provides illustrative quotes.

For peer review only - http://bmjopen.bmj.com/site/about/guidelines.xhtml

Table 3 Participant quotes from qualitative interviews

**Acceptability**<br>"It is ten weeks, you do it twice a week. Personally for the first say three or four weeks, I'd think well this is getting me nowhere, but then you think that you notice things, things are improving and at the end of ten weeks you want to go for twenty weeks (4:119-125)<br><br>"I'd tell them [another stroke survivor] to go ahead and do it and to take it step by step and not to worry about it. Because you are treated with great respect, it was wonderful and they were. I'll never be able to speak highly enough of them." (25:388-390)<br><br>***Intervention approach***<br>"It opened my eyes to what can be done you know. How can I put it? It wasn't as if I believed that I couldn't do something it was being pointed in the right direction…heh I can do it…Great you've done it, you did it and you do it again. Yeah it was great" (4:358-361). "It wasn't easy at first, but I used to manage it" (5:246)<br><br>"It was the way they addressed how you do your exercises. What it is doing to you and all the rest of it. Now to me that was absolutely important, because it made sense of why you are doing all this pumping up and down, and if you can't do that, do this." (22:252-255)<br><br>"It was you felt as if you were a human being with them. You know and you were treated with respect…and although you couldn't do things and you felt a bit of an idiot, they never let you feel like that" (25:567-572).<br><br>"It's a bit like playing scales…it's not creative but as I gradually realise it, it could potentially be creative…doing something that I had been doing without thinking before and now couldn't. …Now and again I walk without my stick without realising it, that's creative I think." (6:354-392)<br><br>***Impact of programme - psychologically***<br>"I suppose it is attitude of mind as much as anything. I mean I felt I'd gone through that stage of training and that I was going to get better. It built my spirit up…I felt as if it, well it was worth the three months you know and at the end of the day I hope I'm going to get back to something like normal" (16:358-365)<br><br>"It really helped me mentally, you know I thought right I can do this because before I was going into my shell, thinking I can't do this and I can't do that. Oh I am not going out. Then I went on that [ReTrain] and it gave me an element of confidence." (43:562-565).<br><br>***Impact of programme – physically***<br>"you started to notice they are actually starting to fall into place. I don't remember doing that last time. But I am doing it now great get on with it I am doing it faster now" (4:189-190)<br>"I know if I went down which I did one day in the hall in the early stages of coming back home and I did manage to get up and walk upstairs…but I wouldn't have been able to do that had I not had that [training]" (16:475-477)<br><br>***Homework adherence***<br>"trainers were always on about doings exercises at home…I could never pin him down to how long that should be for though" (6; line 624).<br><br>***Programme technique adherence***<br>"I think that was the big thing you saw the benefits after the second, well the first or second session we had. 'Oh we can do

a) Study Information

Participants considered information received as adequate. Five noted that information was limited, but most were unconcerned. Two added that too much information may have been detrimental to recruitment. Four others were satisfied with the information they received.

b) Outcome Measure Burden

Participants found the assessment process acceptable. Fifteen indicated no burden. Three participants indicated that they needed help from their carers to complete questionnaires,

For peer review only - http://bmjopen.bmj.com/site/about/guidelines.xhtml

particularly recalling and reporting health resource use, placing a time burden on their carer.

c) Venue

Half of the ReTrain participants were very positive about the training venues. Important features were: space, provision of fluids (water, tea), easy availability of parking. For some the travelling distance was a concern; two noted their venue (a gym) was very noisy, insufficiently heated and the session time was too early. Some noted the small amount of equipment as an advantage (it aided transfer of exercises to their home) whereas others felt the equipment was not sufficiently specialist.

d) Adherence to ReTrain (see also Objective 5)

All ten ReTrain interviewees reported training in the five core (ARNI) techniques. Homework was discussed by all but lacked specificity, only two had clear homework examples that were effectively incorporated into their training. Although goal setting was a core element, only four specifically identified how their goals were linked into their overall programme. Three participants reported not attending drop-in sessions due to lack of information. Of three who attended two suggested the drop-ins repeated previous sessions.

e) Group dynamics

Group working was positively regarded and seen as integral to programme effectiveness. There were exceptions, one participant did not find 'performing' in public a positive experience. Likewise some suggested that groups reduced training intensity relative to one-to-one training.

f) Co-morbidities

Participants identified several co-morbidities e.g.: knee replacements, cancer, angina, diabetes, amputation and depression. These had potential to impact on both the training and research participation but for most any concerns were accommodated by trainers. However, in one case some uncomfortable discussions occurred before an appropriate balance of perceived capability and training challenges was reached. Three participants with visual deficits, dyslexia and dysgraphia mentioned difficulties completing the research documents.

g) Carer Health

Two ReTrain participants commented on how commitment to the programme impacted on their partner's health: one stopped attending sessions because the time away resulted in excessive strain on his wife; another expressed similar concern but did not stop attending.

h) Trainer Manual

For peer review only - http://bmjopen.bmj.com/site/about/guidelines.xhtml

We refined the Trainer Manual throughout the study. Issues raised during interviews guided revisions including greater emphasis and clarification about use of goal setting, drop-ins, homework diaries, and managing participants with co-morbidities.

Objective 4: *Assess outcome completion and burden*

We collected baseline (n=41), 6-month (n=40) and 9-month (n=41) follow-up data on the majority of participants (Figure 2). Accelerometry wear time (24 hours for 7 days) was high, most having 6 or more valid days (≥ 16hrs per day, including ≥ 1 weekend day). Only two participants at baseline, one at 6-months and three at 9-months did not achieve 4 valid days of wear time. There was very little missing data. For three primary outcome measures there was only one participant with missing data at any given assessment time-point. For secondary outcomes there was either no missing data or only one to two participants with missing data at each time-point, apart from the exercise diary (between two and four participants with missing data at each time-point) and the Service Receipt Inventory (between three and seven participants with missing data). There were eight participants without accelerometer data at 9-month assessment owing to hardware (device) and software (data extraction method) malfunctions.

Objective 5: *Perform process evaluation with an assessment of intervention fidelity*

We implemented a comprehensive video recording schedule (over 200 recordings) to capture participant and trainer adherence to key ARNI techniques. Both trainers and participants demonstrated high adherence. Modifications to techniques (to accommodate participant co-morbidities) were captured and informed Trainer Manual development.

We combined metrics from attendance registers and homework records to generate a ‘dose’/adherence score, categorising individuals into low (< 50%), medium (50% - 75%) and high (>75%) adherence categories. Of 23 ReTrain participants two did not receive the intervention (one returned to work; one withdrew from study), five had low adherence, five medium adherence, and eleven high adherence. These latter 16 (70%) were considered to have received sufficient ‘dose’ of ReTrain.

Trainers varied in their completion of session checklists: pre-exercise and end-of-session components were less consistently reported compared to ARNI techniques but overall there was good adherence to programme delivery.

Objective 6: *Calculate the cost of intervention delivery and feasibility of collecting health and social service resource use.*

ReTrain costs were generated for each cohort, accounting for different programme sizes (four or eight participants) and venues. Costs per participant ranged from £615 to £972. The total per participant cost for ReTrain (assuming 24 participants) was £777. We conducted medical notes review on 35/41 participants and compared this ‘gold standard’ with self-reported health resource use. Participants reported using fewer resources compared to case

For peer review only - http://bmjopen.bmj.com/site/about/guidelines.xhtml

notes review. Data from medical notes informed the cost-utility and effectiveness frameworks for use in a definitive trial.

Descriptive analysis of participant outcomes

Primary and secondary outcome data are summarised in Table 4a and 4b respectively.

For peer review only

For peer review only - http://bmjopen.bmj.com/site/about/guidelines.xhtml

Table 4a: Number, means and standard deviations as a function of trial arm and measurement time point for candidate primary outcome measures in the ReTrain pilot trial

	Data collection time point					
	Baseline		6 month^		9 month^	
**Measures:** n, Mean (SD)	**ReTrain (N=23)**	**Control (N=22)**	**ReTrain (N=21)**	**Control (N=20)**	**ReTrain (N=21)**	**Control (N=20)**
Rivermead Mobility Index	23, 11.41 (3.05)	22, 11.68 (2.23)	21, 12.14 (2.73)	19, 12.47 (1.87)	21, 12.24 (3.27)	20, 12.65 (1.81)
Modified Patient Specific Functional Scale	22, 2.95 (1.85)	22, 2.55 (1.23)	21, 3.47 (2.12)	19, 3.56 (1.69)	21, 3.25 (2.03)	20, 3.74 (1.86)
Timed Up and Go (secs) [a]	23, 27.57 (27.57)	21, 21.24 (11.18)	21, 20.76 (19.64)	19, 16.37 (9.69)	21, 20.76 (19.25)	20, 15.95 (12.00)
Physical Activity: **Diary***	21, 6.67 (19.20)	20, 10.69 (17.39)	21, 17.39 (24.28)	19, 25.60 (34.98)	19, 13.92 (22.25)	20, 35.11 (49.70)
Physical Activity (**Accelerometer): Total PA minutes***[b]	21, 145.10 (118.27)	20, 165.56 (139.09)	19, 134.88 (129.77)	18, 178.15 (155.07)	16, 152.08 (118.52)	17, 197.42 (144.31)
Physical Activity **(Accelerometer): Light PA minutes*** [b]	21, 92.78 (93.15)	20, 110.18 (115.65)	19, 95.67 (105.50)	18, 121.80 (122.46)	16, 99.33 (99.54)	17, 134.54 (126.36)
Physical Activity **(Accelerometer): MVPA PA minutes*** [b]	21, 52.32 (68.94)	20, 55.38 (39.66)	19, 39.21 (39.33)	18, 56.35 (51.26)	16, 52.75 (60.03)	17, 62.88 (41.73)
Physical Activity **(Accelerometer): Moderate PA minutes*** [b]	21, 50.53 (66.77)	20, 53.38 (37.04)	19 37.73 (37.40)	18, 53.07 (43.99)	16, 51.11 (58.54)	17, 60.93 (40.84)
Physical Activity **(Accelerometer): Vigorous PA minutes*** [b]	21, 1.79 (3.85)	20, 2.00 (3.96)	19, 1.48 (2.39)	18, 3.28 (8.14)	16, 1.64 (2.38)	17, 1.94 (2.33)

Note: ^ post randomisation; *Average minutes of physical activity per day; [a]Precision to 10 ms; [b]Measurement recorded 100 times a second (accelerometer set to a sampling frequency of 100 Hz).

For peer review only

For peer review only - http://bmjopen.bmj.com/site/about/guidelines.xhtml

Table 4b: Number, means and standard deviations as a function of trial arm and measurement time point for candidate secondary outcome measures in the ReTrain pilot trial

	Data collection time point					
	Baseline		6 month^		9 month^	
**Measures:** n, Mean (SD)	**ReTrain (N=23)**	**Control (N=22)**	**ReTrain (N=21)**	**Control (N=20)**	**ReTrain (N=21)**	**Control (N=20)**
Fatigue Assessment Scale	23, 27.17 (7.14)	22, 25.49 (7.44)	21, 24.05 (6.26)	19, 24.05 (8.86)	20, 27.45 (5.95)	20, 25.83 (9.14)
Stroke Self-efficacy Questionnaire	22, 72.41 (22.00)	22, 73.46 (17.87)			20, 73.73 (19.63)	20, 74.40 (16.94)
Outcome expectations for exercise Scale (Exercise Beliefs)	23, 3.66 (0.70)	22, 3.78 (0.52)			19, 4.03 (0.59)	19, 3.73 (0.52)
Short self-efficacy for Exercise Scale (Exercise self-efficacy)	23, 3.26 (0.92)	22, 3.32 (0.89)			19, 3.32 (0.89)	18, 3.22 (1.06)
Stroke Quality of Life Scale(Total)	22, 3.31 (0.68)	22, 3.45 (0.69)			20, 3.38 (0.70)	20, 3.63 (0.82)
EQ-5D-5L	22, 0.51 (0.25)	20, 0.55 (0.24)			19, 0.52 (0.24)	20, 0.62 (0.25)
SF-12: Physical Component	21, 33.12 (7.22)	20, 31.83 (6.69)			19, 33.74 (6.44)	19, 33.25 (6.91)
SF-12: Mental Component	21, 50.10 (7.11)	20, 50.68 (7.98)			19, 50.47 (6.51)	19, 48.05 (8.45)
Modified Caregiver Strain Index (Carer Burden)	8, 11.39 (8.03)	10, 7.40 (7.83)			9, 9.89 (7.22)	6, 9.50 (8.92)

Note: ^ post randomisation; grey cells indicate measurement not taken at this time point

For peer review only - http://bmjopen.bmj.com/site/about/guidelines.xhtml

Table 4a and 4b report mean scores across, respectively, candidate primary and secondary outcome measures at each time point of the pilot trial. The trial was not powered to detect differences in outcome between trial arms or over time and so we do not interpret the patterns of means. However, the results clearly demonstrate that we were able to collect the necessary data and retained acceptable completion rates on all measures across all time points of the study. Attrition was lower than the 20% expected. For each outcome measure (except carer burden as not everyone had a carer) we achieved in excess of the 30 cases (i.e. 15 completed measurements per arm) recommended for pilot studies to estimate outcome variance.

Safety

During assessment periods there was one serious but unrelated event in the intervention group (none in the control group) and slightly fewer overall adverse events in the intervention group (Table 5a).

Table 5a: Adverse Events (AE) and Serious Adverse Events (SAE) reported during 6 and 9-month outcome assessment periods for both ReTrain and Control group

	Event Type	Total Events	Attribution				N People Reporting Event
ReTrain (N=21)			Related	Probably Related	Possible Related	Unrelated	
	**AE**	125[a]	6	5	73	41	19
	**SAE**	1[b]	0	0	0	1	1
Control (N=20)	Event Type	Total Events	Related	Probably Related	Possible Related	Unrelated	N People Reporting Event
	**AE**	150[c]	0	0	0	150	19
	**SAE**	0	0	0	0	0	0

[a]: Muscle soreness (n=26); fatigue (n=58); falls (n=12); trips (n=10); other (n=19; including but not limited to: low mood, itchiness, colds, issues with eyesight, cystitis).

[b]: Ambulance conveyance to A&E due to reaction to antibiotics being taken for chest infection.

[c]: Muscle soreness (n=39); fatigue (n=50); falls (n=19); trips (n=12); other (n=30; including but not limited to: low mood, depression, dizzy spells, sore toes, poor memory, colds, poor sleep, loss of sense of smell, issues with eyesight).

For peer review only

Table 5b: Adverse Events (AE) and Serious Adverse Events (SAE) reported during ReTrain programme

	Event Type	Total Events	Attribution				N People Reporting Event
ReTrain (N=21)			Related	Probably Related	Possible Related	Unrelated	
	**AE**	2[a]	7	0	12	3	11
	**SAE**	6[b]	0	1	1	4	5

[a]: Muscle soreness (n=0); fatigue (n=2); falls (n=10); trips (n=1); other (n=9; including but not limited to: fainting; twisted or swollen ankle, suspected TIA (non-confirmed)).

[b]: Urine retention (n=3); black-out/fainted (n=1); renal & heart failure (n=1); TIA (n=1).

For ReTrain only (Table 5b) there were six serious adverse events during the intervention period: four were unrelated, one possibly related (fainted) and one probably related (TIA) to the intervention. Of the 22 adverse events reported, three of them occurred at the venue (1 x fall; 1 x trip; 1 x ankle strain).

Discussion

The ReTrain pilot trial met all its pre-stated feasibility objectives: the intervention, trial design and research processes were acceptable to participants as well as feasible and safe to deliver; we demonstrated feasibility of recruitment (recruiting above our target of 48), and retention (less than 20% attrition). At the point of randomisation we were slightly under target (45/48). However due to high retention we preserved the number of datasets required (30) to calculate sample size estimates. Furthermore, participants were not unduly burdened by study requirements and there were high completion rates for most outcome measures. We also successfully rehearsed procedures for process and health economic evaluations as well as trial governance processes (trial management and independent trial steering meetings) and maintained our strong Patient and Public Involvement. Participant interviews, outcome measurement results and fidelity assessments highlighted refinements that we have already, or can, put in place for a future definitive RCT of ReTrain. For example, we have some new insights into how to enhance delivery by trainers and engagement by participants (e.g. by placing more focus on individually tailored goal setting; stressing goal and homework reviews; better explanation and promotion of the drop-in sessions). These are all relatively small amendments that are likely to enhance the impact of the training programme. Our trial compares favourably with another feasibility RCT assessing the delivery of the Bridges stroke self-management programme[44] which had relatively low recruitment, questions regarding programme delivery in addition to usual

For peer review only - http://bmjopen.bmj.com/site/about/guidelines.xhtml

rehabilitation, and recommendations for further assessment of intervention fidelity. Some of their findings were similar to ReTrain: participants were broadly positive about their programme; health professionals found it acceptable to use and researchers noted the lack of outcome measure sensitivity for detecting change[44].

Limitations and lessons for planning design of a future trial

When planning this study we selected our candidate primary outcome measures on the basis that they were likely to measure improvements that could be attributed to our intervention; our pilot work was therefore to determine acceptability and feasibility (including their psychometric utility) of these measures. However we were not able to identify a clear candidate primary outcome for a definitive RCT from this pilot work. It is possible that an 'activities of daily living' measure (as typically used in rehabilitation studies) may be more useful in a future definitive trial. Identifying robust outcome measures in rehabilitation trials is a common problem[45] compounded by variability in stroke related disability and participants' comorbidities. This pilot trial was not designed (statistically powered) to test for differences between treatment arms, so no inferential analyses were performed. Any perceived trend (or absence of a trend) should not be interpreted as an indication of an effect (or its absence) and outcomes should not be selected based upon any assumed trend. Acceptability outcomes coupled with a pragmatic and efficient (cost-effective) trial design better inform choice of outcome. From our sample the Timed Up and Go task would be unsuitable due to potentially large sample size requirements (~2000 participants) and the baseline high levels of mobility meant the Rivermead Mobility Index demonstrated a ceiling effect, so could only be used if we altered inclusion criteria. Physical activity was measured robustly via accelerometry and may be the best candidate. We had some software and hardware malfunctions but important lessons have been learned to mitigate these problems in future. Capture of frequency and intensity of activity would allow comparison with stroke guidelines. Although there is a cost implication, accelerometry provides a more objective measurement of daily activity and may also be an adequate proxy of functional mobility, however we will also investigate the benefits of using other PA measures such as questionnaires (instead of our diaries) or using multiple measures such as accelerometry and heart rate monitors whilst being aware of problems with compliance and participant burden[46].

Further limitations relate to the lack of validation of our adherence measure and the local demographics: our sample did not have a wide age range or ethnic diversity. Whilst we did demonstrate delivery in different locations in the South West our plans for a larger definitive trial would include a wider demographic from more centres across the UK.

For a future trial we plan to implement more readable, higher quality written (and pictorial) information and questionnaires although the amount of information provided was appropriate. We will mitigate recruitment loss prior to randomisation by establishing expression of interest and eligibility to take part but delaying taking consent until we are

For peer review only - http://bmjopen.bmj.com/site/about/guidelines.xhtml

confident of sufficient numbers to create a cohort for randomisation; this has resource implications that will need to be built into future funding. We will run ReTrain in community centres or halls as these were more acceptable and much cheaper than gyms; we will provide a more detailed ReTrain induction to ensure trainers understand and communicate all components of the programme. For the QALY comparisons recent policy changes mean the conversion from SF-12 to SF-6D has been phased out, and so less justification for using the SF-12 in a future study. Instead we will consider using the Stroke Impact Scale (SIS) as this is a valid health-related QoL measure. This may also be a better candidate self-report primary outcome measure for a definitive trial as it has shown sensitivity in long-term stroke survivors who have mild to moderate stroke[47]. The SIS assesses multiple facets of physical and emotional issues and so would align with perceived physical and psychological benefits participants attribute to ReTrain. Our sample size estimates for candidate objective primary outcomes (Table 2) indicate we will need a moderately sized trial (n=562, effect size 0.3 or n=413, effect size 0.35) for PA assessed by accelerometry or a smaller trial (n=96) if we use the physical component domain of the SIS (based on 80% power, 5% alpha and assuming 20% attrition[48]). We have established appropriate process evaluation methods to capture multiple facets of intervention fidelity.

Generalisability

This pilot study was not designed to demonstrate generalisability however our participant population represent the sub-set of community-dwelling stroke survivors who have some independent mobility but remain with stroke-related disability that affects their QoL. Our participants also represent the growing proportion of people who have more than one long-term condition. ReTrain techniques target the effects of stroke but can accommodate other conditions which trainers take into account when preparing the participant's individually tailored programme. Some of the key ReTrain (ARNI based) techniques are designed for people with unilateral impairment, such as hemiparesis; however one of our participant's main unilateral impairment was due to diabetes related lower limb amputation, illustrating how ReTrain can accommodate people with multiple co-morbidities.

Conclusion

Our pilot trial has demonstrated that ReTrain is feasible, acceptable, and safe. We met our recruitment and retention targets and demonstrated our ability to run our intervention in different locations. Participants were not unduly burdened by study requirements and most outcome measures had high levels of completion. We successfully tested procedures for process and health economic evaluations. Participant interviews, outcome measurement results and fidelity assessments highlighted some issues needing refinement prior to a future definitive RCT of ReTrain. Many of these have already been addressed and we intend to seek funding for a definitive trial.

For peer review only - http://bmjopen.bmj.com/site/about/guidelines.xhtml

Other information

Protocol Version: 5 Date: 20/04/2016. Published version available here: http://bmjopen.bmj.com/content/6/10/e012375.full

Acknowledgements

We thank our funders, the Stroke Association and the Peninsula Patient Involvement Group with the ReTrain Stroke Service User Group for their help. The National Institute for Health Research (NIHR) Collaboration for Leadership in Applied Health Research and Care South West Peninsula at the Royal Devon and Exeter NHS Foundation Trust also supported this work but views expressed are those of the author(s) and not necessarily those of the NHS, the NIHR or the Department of Health. We also thank our Trial Steering Committee: Ailie Turton (University of the West of England) Siobhan Creanor (Plymouth University), Debbie Neal (Bournemouth University), Justin Smallwood (Patient and Public representative), and Gail Seymour (University of Exeter - Sponsor).

Ethical review by NRES Committee South West – Cornwall & Plymouth (REC ref: 15/SW/04).

Author's Contributions

SGD led the team and drafted this article, RC prepared protocol, ethical submission and amendments, managed the project, contributed to analysis; LP drafted protocol prior to funding application, conducted interviews, contributed to analysis, AF, MJ, RA, MN, SGD & LP provided stroke rehabilitation expertise; RST provided statistical and trial methodological expertise, led analysis; MN provided qualitative expertise and analysed qualitative data; AIS led accelerometry work, supported by RP who provided accelerometry analysis; SGD & LP provided process evaluation expertise, SGD led the process evaluation and supervised LH; LH led video analysis work; AS provided health economic expertise and led economic work supported by PL; SS provided patient and public involvement expertise. All authors commented on the manuscript.

Data sharing statement: Participants did not consent for datasets to be stored or accessed outside of the research team. Therefore no datasets have been made publicly available.

Competing Interests: We declare funding from the Stroke Association.

For peer review only - http://bmjopen.bmj.com/site/about/guidelines.xhtml

References

1. Feigin VL, Barker-Collo S, McNaughton H, et al. Long-term neuropsychological and functional outcomes in stroke survivors: current evidence and perspectives for new research. *Int J Stroke* 2008;3(1):33-40.
2. Townsend N, Wickramasinghe K, Bhatnagar P, et al. Coronary heart disease statistics: 2012 edition. London: British Heart Foundation, 2012.
3. Teasell R, Mehta S, Pereira S, et al. Time to rethink long-term rehabilitation management of stroke patients. *Top Stroke Rehabil* 2012;19(6):457-62.
4. McKevitt C, Fudge N, Redfern J, et al. Self-reported long-term needs after stroke. *Stroke* 2011;42(5):1398-403.
5. Intercollegiate Stroke Working Party. National Clincial Guideline for Stroke, Fifth Edition. 5 ed. London: *Royal College of Physicians* 2016.
6. Rand D, Eng JJ, Tang PF, et al. How active are people with stroke?: use of accelerometers to assess physical activity. *Stroke* 2009;40(1):163-8.
7. Stroke Association. Stroke Statistics. London: Stroke Association, 2013.
8. Best C, van Wijck F, Dennis J, et al. A survey of community exercise programmes for stroke survivors in Scotland. *Health Soc Care Community* 2012;20(4):400-11.
9. NHS Stroke Improvement Programme. Life after stroke: Participating in community exercise and fitness 2012 [Available from: http://www.improvement.nhs.uk/stroke/CommunityStrokeResource/CSRLifeafterstroke/CSRLifeafterstrokeexercisepoststroke/tabid/226/Default.aspx accessed July 2012.
10. Harrington R, Taylor G, Hollinghurst S, et al. A community-based exercise and education scheme for stroke survivors: a randomized controlled trial and economic evaluation. *Clin Rehabil* 2010;24(1):3-15.
11. French B, Thomas LH, Leathley MJ, et al. Repetitive task training for improving functional ability after stroke. *Cochrane Database Syst Rev* 2007(4):Cd006073.
12. Lennon S, McKenna S, Jones F. Self-management programmes for people post stroke: a systematic review. *Clin Rehabil* 2013;27(10):867-78.
13. Brazzelli M, Saunders David H, Greig Carolyn A, et al. Physical fitness training for stroke patients. *Cochrane Database Syst Rev* 2011; (11). http://www.mrw.interscience.wiley.com/cochrane/clsysrev/articles/CD003316/frame.html.
14. Balchin T. The Successful Stroke Survivor: A new guide to functional recovery from stroke. Lingfield, UK: ARNI Trust 2011.
15. Poltawski L. Survey of Group ARNI classes in England, 2011, unpublished report.
16. Craig P, Dieppe P, Macintyre S, et al. Developing and evaluating complex interventions: the new Medical Research Council guidance. *BMJ* 2008;337:a1655.
17. Poltawski L, Abraham C, Forster A, et al. Synthesising practice guidelines for the development of community-based exercise programmes after stroke. *Implement Sci* 2013;8:115.
18. Kilbride C, Norris M, Theis N, et al. Action for Rehabilitation from Neurological Injury (ARNI): A pragmatic study of functional training for stroke survivors. *Open J Ther Rehabil* 2013;1:40-51.
19. Poltawski L, Briggs J, Forster A, et al. Informing the design of a randomised controlled trial of an exercise-based programme for long term stroke survivors: lessons from a before-and-after case series study. *BMC Res Notes* 2013;6:324.

For peer review only - http://bmjopen.bmj.com/site/about/guidelines.xhtml

20. Norris M, Kilbride C, Mohagheghi A, et al. A Qualitative Exploration of Participation in an Exercise Instructor Led Functional Training Programme for Community Dwelling Stroke Survivors. *Int J Ther Rehabil* 2013;20(12):597-605.
21. Eldridge SM, Chan CL, Campbell MJ, et al. CONSORT 2010 statement: extension to randomised pilot and feasibility trials. *BMJ* 2016;355.
22. Dean SG, Poltawski L, Forster A, et al. Community-based Rehabilitation Training after stroke: protocol of a pilot randomised controlled trial (ReTrain). *BMJ Open* 2016;6(10).
23. Stroke Association. Exercise and stroke London2013 [Available from: https://www.stroke.org.uk/sites/default/files/exercise_and_stroke.pdf accessed 24 05 2017 2017].
24. ACSM. American College of Sports Medicine Guidelines for Exercise Testing and Prescription. Philadelphia: American College of Sports Medicine, 2005.
25. Carnes D, Mullinger B, Underwood M. Defining adverse events in manual therapies: A modified Delphi consensus study. *Man Ther* 2010;15(1);2-6.
26. Green J, Forster A, Young J. A test-retest reliability study of the Barthel Index, the Rivermead Mobility Index, the Nottingham Extended Activities of Daily Living Scale and the Frenchay Activities Index in stroke patients. *Disabil Rehabil* 2001;23(15):670-6.
27. Hsieh CL, Hsueh IP, Mao HF. Validity and responsiveness of the rivermead mobility index in stroke patients. *Scand J Rehabil Med* 2000;32(3):140-2.
28. Ng SS, Hui-Chan CW. The timed up & go test: its reliability and association with lower-limb impairments and locomotor capacities in people with chronic stroke. *Arch Phys Med Rehabil* 2005;86(8):1641-7.
29. Stratford P, Gill C, Westaway M, et al. Assessing Disability and Change on Individual Patients: A Report of a Patient Specific Measure. *Physiother Can* 1995;47(4):258-63.
30. Jones F, Partridge C, Reid F. The Stroke Self-Efficacy Questionnaire: measuring individual confidence in functional performance after stroke. *J Clin Nurs* 2008;17(7b):244-52.
31. Michielsen HJ, De Vries J, Van Heck GL. Psychometric qualities of a brief self-rated fatigue measure: The Fatigue Assessment Scale. *J Psychosom Res* 2003;54(4):345-52.
32. Mead G, Lynch J, Greig C, et al. Evaluation of fatigue scales in stroke patients. *Stroke* 2007;38(7):2090-5.
33. Shaughnessy M, Resnick BM, Macko RF. Reliability and validity testing of the short self-efficacy and outcome expectation for exercise scales in stroke survivors. *J Stroke Cerebrovasc Dis* 2004;13(5):214-9.
34. Bohannon RW, Maljanian R, Lee N, et al. Measurement properties of the short form (SF)-12 applied to patients with stroke. *Int J Rehabil Res* 2004;27(2):151-4.
35. Herdman M, Gudex C, Lloyd A, et al. Development and preliminary testing of the new five-level version of EQ-5D (EQ-5D-5L). *Qual Life Res* 2011;20(10):1727-36.
36. Williams LS, Weinberger M, Harris LE, et al. Development of a stroke-specific quality of life scale. *Stroke* 1999;30(7):1362-9.
37. Thornton M, Travis SS. Analysis of the reliability of the modified caregiver strain index. *J Gerontol B, Psychol Sci Soc Sci* 2003;58(2):S127-32.
38. Craig LE, Wu O, Bernhardt J, et al. Approaches to economic evaluations of stroke rehabilitation. *Int J Stroke* 2014;9(1):88-100.
39. Browne RH. On the use of a pilot sample for sample size determination. *Stat Med* 1995;14(17):1933-4.

For peer review only

40. Bruno A, Akinwuntan AE, Lin C, et al. Simplified Modified Rankin Scale Questionnaire: Reproducibility Over the Telephone and Validation With Quality of Life. *Stroke* 2011; 42(8)2276-9.
41. Hislop J, Adewuyi TE, Vale LD, et al. Methods for specifying the target difference in a randomised controlled trial: the Difference ELicitation in TriAls (DELTA) systematic review. *PLoS Med* 2014;11(5):e1001645.
42. Gautschi OP, Stienen MN, Corniola et al. Assessment of the Minimum Clinically Important Difference in the Timed Up and Go Test After Surgery for Lumbar Degenerative Disc Disease. *Neurosurgery* 2017; 80 (3):380-385.
43. Horn KK, Jennings S, Richardson G, et al. The patient-specific functional scale: psychometrics, clinimetrics, and application as a clinical outcome measure. *J Orthop Sports Phys Ther* 2012; 42 (1):30-42.
44. McKenna S, Jones F, Glenfield P, et al. Bridges self-management program for people with stroke in the community: A feasibility randomized controlled trial. *Int J Sroke* 2015;10(5):697-704.
45. Wade DT. Outcome measures for clinical rehabilitation trials: impairment, function, quality of life, or value? *Am J Phys Med Rehabil* 2003;82(10 Suppl):S26-31.
48. Mansfield A, Knorr S, Poon V, et al. Promoting Optimal Physical Exercsie for Life: An Exercise and Self-Management Program to Encourage Participation in Physical Activity after Discharge from Stroke Rehabilitation – A Feasibility Study. *Stroke Research and Treatment* 2016; [Available from http://dx.doi.org/10.1155/2016/9476541].
47. Rehabilitation Institute of Chicago. Rehabilitation Measures Database - Stroke Impact Scale [Available from: http://www.rehabmeasures.org/Lists/RehabMeasures/DispForm.aspx?ID=934 accessed 21 06 2017].
48. Saywell N, Vandal AC, Brown P, et al. Telerehabilitation to improve outcomes for people with stroke: study protocol for a randomised controlled trial. *Trials* 2012;13(1):233.

For peer review only - http://bmjopen.bmj.com/site/about/guidelines.xhtml

Figure 1 – Recruitment and randomisation by cohort

For peer review only

For peer review only - http://bmjopen.bmj.com/site/about/guidelines.xhtml

Figure 2 – Participant flow through the trial

For peer review only

For peer review only - http://bmjopen.bmj.com/site/about/guidelines.xhtml

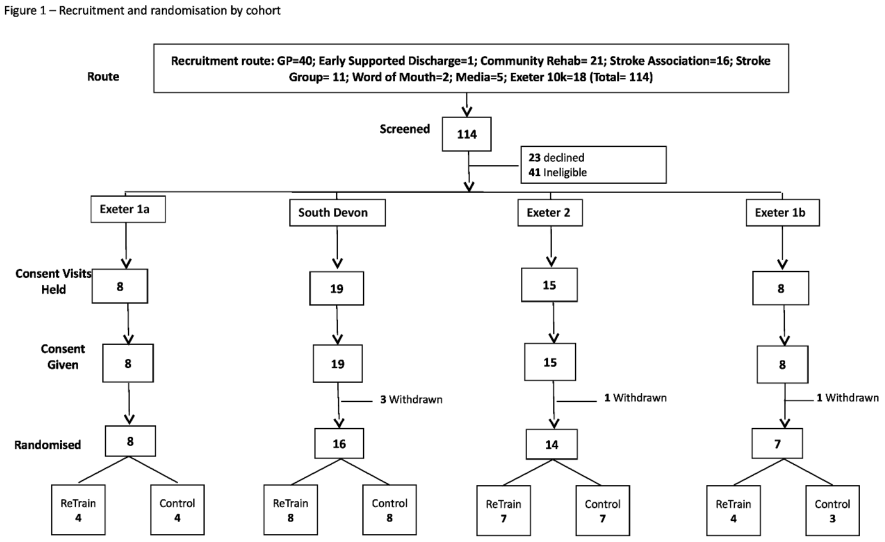

Figure 1 – Recruitment and randomisation by cohort

297x209mm (300 x 300 DPI)

For peer review only

Figure 2 – Participant flow through the trial

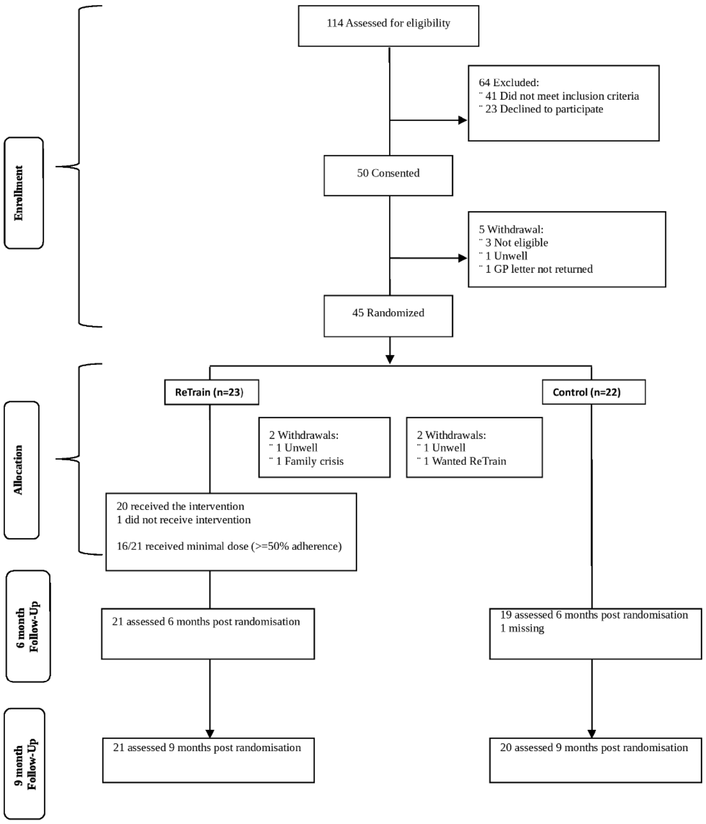

Figure 2 – Participant flow through the trial

209x297mm (300 x 300 DPI)

Table 2 | CONSORT checklist of information to include when reporting a pilot trial

Section/topic and item No	Standard checklist item	Extension for pilot trials	Page No where item is reported
**Title and abstract**			
1a	Identification as a randomised trial in the title	Identification as a pilot or feasibility randomised trial in the title	1
1b	Structured summary of trial design, methods, results, and conclusions (for specific guidance see CONSORT for abstracts)	Structured summary of pilot trial design, methods, results, and conclusions (for specific guidance see CONSORT abstract extension for pilot trials)	2
**Introduction**			
Background and objectives:			
2a	Scientific background and explanation of rationale	Scientific background and explanation of rationale for future definitive trial, and reasons for randomised pilot trial	4
2b	Specific objectives or hypotheses	Specific objectives or research questions for pilot trial	4-5
**Methods**			
Trial design:			
3a	Description of trial design (such as parallel, factorial) including allocation ratio	Description of pilot trial design (such as parallel, factorial) including allocation ratio	5
3b	Important changes to methods after trial commencement (such as eligibility criteria), with reasons	Important changes to methods after pilot trial commencement (such as eligibility criteria), with reasons	N/A
Participants:			
4a	Eligibility criteria for participants		5
4b	Settings and locations where the data were collected		5-6
4c		How participants were identified and consented	5
Interventions:			
5	The interventions for each group with sufficient details to allow replication, including how and when they were actually administered		5-6
Outcomes:			
6a	Completely defined prespecified primary and secondary outcome measures, including how and when they were assessed	Completely defined prespecified assessments or measurements to address each pilot trial objective specified in 2b, including how and when they were assessed	6
6b	Any changes to trial outcomes after the trial commenced, with reasons	Any changes to pilot trial assessments or measurements after the pilot trial commenced, with reasons	N/A
6c		If applicable, prespecified criteria used to judge whether, or how, to proceed with future definitive trial	N/A
Sample size:			
7a	How sample size was determined	Rationale for numbers in the pilot trial	6-7
7b	When applicable, explanation of any interim analyses and stopping guidelines		N/A
Randomisation:			
Sequence generation:			
8a	Method used to generate the random allocation sequence		7
8b	Type of randomisation; details of any restriction (such as blocking and block size)	Type of randomisation(s); details of any restriction (such as blocking and block size)	7
Allocation concealment mechanism:			
9	Mechanism used to implement the random allocation sequence (such as sequentially numbered containers), describing any steps taken to conceal the sequence until interventions were assigned		7
Implementation:			
10	Who generated the random allocation sequence, enrolled participants, and assigned participants to interventions		7
Blinding:			
11a	If done, who was blinded after assignment to interventions (eg, participants, care providers, those assessing outcomes) and how		7
11b	If relevant, description of the similarity of interventions		N/A
Analytical methods:			
12a	Statistical methods used to compare groups for primary and secondary outcomes	Methods used to address each pilot trial objective whether qualitative or quantitative	7
12b	Methods for additional analyses, such as subgroup analyses and adjusted analyses	Not applicable	N/A

For peer review only - http://bmjopen.bmj.com/site/about/guidelines.xhtml

RESEARCH METHODS AND REPORTING

Table 2 | CONSORT checklist of information to include when reporting a pilot trial

Section/topic and item No	Standard checklist item	Extension for pilot trials	Page No where item is reported
**Results**			
Participant flow (a diagram is strongly recommended):			
13a	For each group, the numbers of participants who were randomly assigned, received intended treatment, and were analysed for the primary outcome	For each group, the numbers of participants who were approached and/or assessed for eligibility, randomly assigned, received intended treatment, and were assessed for each objective	Figure 1a & 1b & P.8
13b	For each group, losses and exclusions after randomisation, together with reasons		Figure 1a & 1b & p.10
Recruitment:			
14a	Dates defining the periods of recruitment and follow-up		8
14b	Why the trial ended or was stopped	Why the pilot trial ended or was stopped	N/A
Baseline data:			
15	A table showing baseline demographic and clinical characteristics for each group		8-10
Numbers analysed:			
16	For each group, number of participants (denominator) included in each analysis and whether the analysis was by original assigned groups	For each objective, number of participants (denominator) included in each analysis. If relevant, these numbers should be by randomised group	Figure 1b
Outcomes and estimation:			
17a	For each primary and secondary outcome, results for each group, and the estimated effect size and its precision (such as 95% confidence interval)	For each objective, results including expressions of uncertainty (such as 95% confidence interval) for any estimates. If relevant, these results should be by randomised group	N/A
17b	For binary outcomes, presentation of both absolute and relative effect sizes is recommended	Not applicable	N/A
Ancillary analyses:			
18	Results of any other analyses performed, including subgroup analyses and adjusted analyses, distinguishing prespecified from exploratory	Results of any other analyses performed that could be used to inform the future definitive trial	10-15
Harms:			
19	All important harms or unintended effects in each group (for specific guidance see CONSORT for harms)		16
19a		If relevant, other important unintended consequences	N/A
**Discussion**			
Limitations:			
20	Trial limitations, addressing sources of potential bias, imprecision, and, if relevant, multiplicity of analyses	Pilot trial limitations, addressing sources of potential bias and remaining uncertainty about feasibility	17-18
Generalisability:			
21	Generalisability (external validity, applicability) of the trial findings	Generalisability (applicability) of pilot trial methods and findings to future definitive trial and other studies	18
Interpretation:			
22	Interpretation consistent with results, balancing benefits and harms, and considering other relevant evidence	Interpretation consistent with pilot trial objectives and findings, balancing potential benefits and harms, and considering other relevant evidence	18-19
22a		Implications for progression from pilot to future definitive trial, including any proposed amendments	19
**Other information**			
Registration:			
23	Registration number and name of trial registry	Registration number for pilot trial and name of trial registry	2, 19
Protocol:			
24	Where the full trial protocol can be accessed, if available	Where the pilot trial protocol can be accessed, if available	19
Funding:			
25	Sources of funding and other support (such as supply of drugs), role of funders		2, 19
26		Ethical approval or approval by research review committee, confirmed with reference number	19

For peer review only - http://bmjopen.bmj.com/site/about/guidelines.xhtml
